# Genome-wide identification and expression analysis of the *VQ* gene family in *Cicer arietinum* and *Medicago truncatula*

**DOI:** 10.7717/peerj.8471

**Published:** 2020-02-04

**Authors:** Lei Ling, Yue Qu, Jintao Zhu, Dan Wang, Changhong Guo

**Affiliations:** College of Life Science and Technology, Harbin Normal University, Harbin, China

**Keywords:** *Cicer arietinum*, *Medicago truncatula*, *VQ* gene family, Gene expression, Abiotic stress, Bioinformatics

## Abstract

Valine-glutamine (VQ) proteins are plant-specific proteins that play crucial roles in plant development as well as biotic and abiotic stress responses. *VQ* genes have been identified in various plants; however, there are no systematic reports in *Cicer arietinum* or *Medicago truncatula*. Herein, we identified 19 and 32 *VQ* genes in *C. arietinum* and* M. truncatula*, respectively. A total of these *VQ* genes were divided into eight groups (I–VIII) based on phylogenetic analysis. Gene structure analyses and motif patterns revealed that these *VQ* genes might have originated from a common ancestor. In silico analyses demonstrated that these *VQ* genes were expressed in different tissues. qRT-PCR analysis indicated that the *VQ* genes were differentially regulated during multiple abiotic stresses. This report presents the first systematic analysis of* VQ* genes from *C. arietinum* and* M. truncatula* and provides a solid foundation for further research of the specific functions of VQ proteins.

## Introduction

Valine-glutamine (*VQ*) genes are plant-specific genes involved in plant growth, development, and various stress responses (Dong et al., 2018; Guo et al., 2018; Jing & Lin, 2015; Cheng et al., 2012; Li et al., 2014). They contain a conserved motif, named the VQ motif, which possesses approximately 50–60 amino acids with a highly conserved FxxhVQxhTG domain (Jing & Lin, 2015; Cai et al., 2019). The mutant strain of *AtVQ14* in the *VQ* domain causes Arabidopsis to produce smaller seeds (Wang et al., 2010a). Recently, *VQ* genes have been identified in multiple plants, such as *Arabidopsis thaliana* (34) (Cheng et al., 2012), *Oryza sativa* (39) (Kim et al., 2013a), *Zea mays* (61) (Song et al., 2016), *Glycine max* (74) (Zhou et al., 2016), and *Vitis vinifera* (18) (Wang et al., 2015). These genes are phylogenetically clustered into different groups, which are dependent on the analysed genomes (Jiang, Sevugan & Ramachandran, 2018). In addition, most VQ proteins reportedly contain fewer than 300 amino acids and lack introns (Jiang, Sevugan & Ramachandran, 2018; Cai et al., 2019).

The VQ proteins have multiple functions at different stages of plant growth (Jiang, Sevugan & Ramachandran, 2018). For example, the *AtVQ8* mutation causes a yellowish-green leaf phenotype and growth retardation throughout the entire developmental period (Cheng et al., 2012). Over-expression of *AtVQ29* reduces the hypocotyl growth of seedlings under special light conditions (Perruc et al., 2004). VQ proteins are also involved in plant responses to biotic and abiotic stresses (Perruc et al., 2004). *AtVQ21 (MKS1)*-overexpressing transgenic plants exhibit decreased resistance to *Botrytis cinerea* but significantly increased resistance to *Pseudomonas syringae* (Lai et al., 2011), and *AtVQ15* (*AtCaMBP25*)-overexpressing transgenic plants exhibit sensitivity to osmotic stress during seed germination and seedling growth (Perruc et al., 2004). In addition, the transcript levels of some *VQ* genes in rice are affected by exposure to drought (Kim et al., 2013a). Most studies have indicated that many VQ proteins interact with WRKY transcription factors, which are not only involved in plant growth but also participate in multiple regulatory pathways (Chen et al., 2018; Lei et al., 2017; Lei, Ma & Yu, 2018; Wang et al., 2015; Yanru et al., 2013; Ye et al., 2016). For instance, AtVQ14 interacts with AtWRKY10 to regulate endosperm growth and seed size (Cheng et al., 2012), and AtVQ9 acts antagonistically with AtWRKY8 to mediate responses to salt stress (Yanru et al., 2013). AtVQ22 could negatively control mediated JA defense through interact with AtWRKY28 and AtWRKY51 (Po et al., 2013). Moreover, MaWRKY26 could physically interact with MaVQ5, restricting the transactivation of the genes which control the JA biosynthetic, indicating that MaVQ5 might act as a repressor of MaWRKY26 in activating the JA biosynthesis in banana (Ye et al., 2016).

Legumes represent the third largest family of seed plants and one of the most important sources of food and nutrition for humans and animals (Wang et al., 2017a; Kim et al., 2013b). *Cicer arietinum* and *Medicago truncatula* are common legumes and model plants that have been used to study legume genomics (Wang et al., 2017a). However, *VQ* genes have not been comprehensively evaluated in *C. arietinum* or *M. truncatula.* In this study, we identified 19 and 32 *VQ* genes in *C. arietinum* and *M. truncatula*, respectively. We conducted a comprehensive analysis to examine their phylogenetic relationship, gene structure, protein motifs, chromosome locations, promoters and collinearity, used silico expression analysis of *VQ* genes to show expression patterns of *VQ* genes in different tissues, and a qRT-PCR analysis to explore their responses to multiple abiotic stresses. This report provides a theoretical basis for the evolutionary relationship and function of the *VQ* genes in *C. arietinum* and *M. truncatula*.

## Materials & Methods

### Identification of *VQ* genes

The current genome sequence and annotation files of *C. arietinum* and *M. truncatula* were downloaded from Phytozome v12.1 (https://phytozome.jgi.doe.gov/pz/portal.html). The most updated Hidden Markov Model (HMM) for the *VQ* gene family (PF05678) was downloaded from the Pfam database (http://pfam.xfam.org) (Punta et al., 2004). We conducted a BLAST search against the entire protein dataset of *C. arietinum* and *M. truncatula* with a cut-off *E*-value of 0.1. Subsequently, all hit protein sequences were extracted using custom Perl scripts. Then, the integrity of the *VQ* domain was evaluated using SMART tools with an e-value <0.1 (Ivica, Tobias & Peer, 2012), and candidate CaVQ and MtVQ proteins composed of a truncated *VQ* domain were identified. Peptide length, molecular weight (MW), and isoelectric point (pI) of each VQ protein were calculated using the online ExPASy program (https://www.expasy.org/) (Wilkins et al., 1999). Detailed information of CaVQ and MtVQ proteins can be found in Table S1.

### Phylogenetic analysis

To investigate the phylogenetic relationships of the *VQ* gene families among *A. thaliana*, *O. sativa*, *C. arietinum*, *M. truncatula,* among them, AtVQ and OsVQ proteins were downloaded from Phytozome v12.1 (http://www.phytozome.org) (Goodstein et al., 2012; Kim et al., 2013a; Cheng et al., 2012). VQ proteins were aligned using the BioEdit program. A neighbour-joining (NJ) phylogenetic tree was constructed for these proteins with MEGA5.0 software (Tamura et al., 2011). Bootstrapping was performed with 1,000 replications. Genes were classified according to their distance homology with *A. thaliana*, *O. sativa*, *G. max* and *P. vulgaris* genes (Cheng et al., 2012; Kim et al., 2013a).

### Motif prediction and gene structure analysis of *VQ* genes

The online MEME analysis was performed to identify unknown conserved motifs (http://meme.ebi.edu.au/) using the following parameters: site distribution: zero or one occurrence (of a contributing motif site) per sequence; maximum number of motifs: 20; and optimum motif width: ≥6 and ≤200 (Bailey et al., 2015) . A gene structure displaying server program (http://gsds.cbi.pku.edu.cn/index.php) was used to display the structures of the *CaVQ* and *MtVQ* genes.

### Chromosomal distribution, gene duplication and collinearity analysis

Physical positions of *CaVQ* and *MtVQ* genes were retrieved from the GFF3 annotation file using a Perl script, and diagrams of their chromosomal locations and duplication events were drawn using MG2C website (http://mg2c.iask.in/mg2c_v2.0/). In addition, gene duplication information was also identified based on public data in the Plant Genome Duplication Data base (PGDD, http://chibba.agtec.uga.edu/duplication/) (Lee et al., 2013). If two homologous genes were separated by five or fewer genes, they were identified as tandem duplications, while if two genes were separated by more than five genes or distributed in different chromosomes, they were referred to as segmental duplications. BLASTP, OrthoMCL (http://orthomcl.org/orthomcl/about.do#release) and Multiple collinear scanning toolkits (MCScanX) with the default parameters were used to analyze the gene replication events (*E* <1 e^−5^, top 5 matches) (Li, Stoeckert & Roos, 2003; Wang et al., 2012).

### Calculating *Ka* and *Ks* of the homologous *VQ* gene pairs

*Ka* and *Ks* were used to assess the selection history and divergence time of gene families (Li, Gojobori & Nei, 1981). The number of synonymous (*Ks*) and nonsynonymous (*Ka*) substitutions of paralogous *MtVQ* gene pairs and orthologous *VQ* gene pairs between *C. arietinum* and *M. truncatula* were computed using the KaKs_Calculator 2.0 with the NG method (Wang et al., 2010b)). Divergence time (*T*) was calculated using the formula *T* = *Ks*/ (2 × 6. 1 × 10^−9^) ×10^−6^ million years ago (MYA) (Wang et al., 2017a).

### Analysis of *cis*-elements in *VQ* promoters

The *cis*-elements of *CaVQ* and *MtVQ* promoters were analysed to further understand the VQ gene family. The 1,500 bp upstream sequences of the CaVQs and MtVQs promoter regions were downloaded FASTA format from the Phytozome database and used to identify the putative *cis*-elements in PlantCARE (http://bioinformatics.psb.ugent.be/webtools/plantcare/html/) (Rombauts et al., 1999).

### In silico expression analysis of *VQ* genes

The transcriptome data in different tissues of *C. arietinum* and *M. truncatula* were available in the NCBI SRA (http://www.ncbi.nlm.nih.gov) with accession numbers PRJNA413872 and PRJNA80163, respectively. The quality-filtered reads were mapped to the respective *C. arietinum* and *M. truncatula* genomes with the spliced read mapper. Clean reads from all samples were mapped to the genome sequence using SAM (Li et al., 2009). TopHat v2.1.0 (Trapnell, Pachter & Salzberg, 2009). Cufflinks v2.1.1 and cuffcompare (Trapnell et al., 2010) were used to estimate the abundance of reads mapped to genes by calculating the fragments per kilobase of transcript per million (FPKM) values. Transcriptome data of the *C. arietinum*, including the nodule, leaf, flower, root, pod and bud; nodule, blade, flower, root, seedpod and bud in *M. truncatula* were obtained. The FPKM (fragment per kilobase per million mapped reads) representing the gene expression level of each *CaVQ* and *MtVQ* was extracted with custom Perl scripts. The heatmaps showing expression profiles were generated using log10-transformed FPKM values. The heatmaps and k-means clustering were generated using R 3.2.2 software (Gentleman et al., 2004).

### Plant material, treatments, RNA extraction and quantitative real-time PCR (qRT-PCR)

*C. arietinum* (ICC4958) and *M. truncatula* (Jemalong A17) were used in this study. ICC4958 is drought tolerant, and Jemalong A17 is salt-sensitive and drought-tolerant. In the greenhouse, seeds were planted in a 3:1 (w/w) mixture of soil and sand, germinated, and irrigated with half-strength Hoagland solution once every 2 days. Seedlings were grown at a night temperature of 18 °C, day temperature of 24 °C, relative humidity of 60%, and 14/10 h photoperiod (daytime: 06:00–20:00). Seedlings that germinated after 8 weeks were subjected to the following environmental conditions: temperatures of 4 (cold) or −8 °C (freezing) and treatment with 300 mM mannitol (drought) and 200 mM NaCl solution (salt). The control (untreated) and treated seedlings were harvested at 1 h, 6 h, 12 h, and 24 h after treatment. All samples were frozen in liquid nitrogen and stored at −80 °C until further use.

Primers were designed to amplify 19 *CaVQ* and 32 *MtVQ* CDS using Primer Express 3.0 software, and the primer pairs are listed in Table S1. Total RNA was extracted from the root of *C. arietinum* and *M. truncatula* using the RNA Prep Pure Plant Kit (Tiangen, Beijing, China). The RNA quality was checked using 1.0% (w/v) agarose gel stained with ethidium bromide (EB) and spectrophotometer analysis and then DNase I treatment was conducted to remove the DNA contaminations (Takara, Shiga-ken, Japan). cDNA was synthesized from total RNA using the ReverTra Ace qPCR RT Kit (Toyobo Life Science, Shanghai, China). Quantitative real-time PCR (qRT-PCR) was performed using SYBR Green and monitored on an ABI 7300 Real-Time PCR system (Applied Biosystems, CA, USA). The PCR conditions were set as follows: 95 °C for 10 min; 40 cycles at 95 ° C for 15 s, 55 °C for 30 s, and 72 ° C for 30 s, a final step to preparation of DNA melting curve at 95 °C for 15 s, and then one cycle at 60 °C for 20 s and one cycle at 95 ° C for 15 s. Rapid detection expression levels of *CaVQ* and *MtVQ* genes using the qRT-PCR with DNA melting curve analysis. The gene *β-actin* was used as a reference gene. The relative expression levels of each gene were analysed using the 2^−^^△△^^Ct^ method (Livak & Schmittgen, 2000). All samples were tested with three technical replicates and three independent biological replicates.

## Results

### Identification and phylogenetic analysis of *VQ* genes in two legumes

A total of 19 and 32 genes putatively encoding *VQ* domains were identified in *C. arietinum* and *M. truncatula*, respectively. We designated the 19 *VQ* genes in *C. arietinum* as *CaVQ1* to *CaVQ19* and 32 *VQ* genes in *M. truncatula* as *MtVQ1* to *MtVQ32* according to their physical locations on the chromosomes (Table 1). The lengths of these VQ proteins ranged from 82 (MtVQ9) to 419 (MtVQ22) amino acids (aa), with an average of 206 aa. Their molecular weights varied from 9.3 (MtVQ9) to 45.8 (CaVQ17/CaVQ19) kDa, and the theoretical isoelectric points (pI) extended from 4.06 (CaVQ4) to 10.68 (MtVQ21) . Among them, MtVQ30 and MtVQ31 as well as CaVQ17 and CaVQ19 were highly similar.

**Figure 1:**
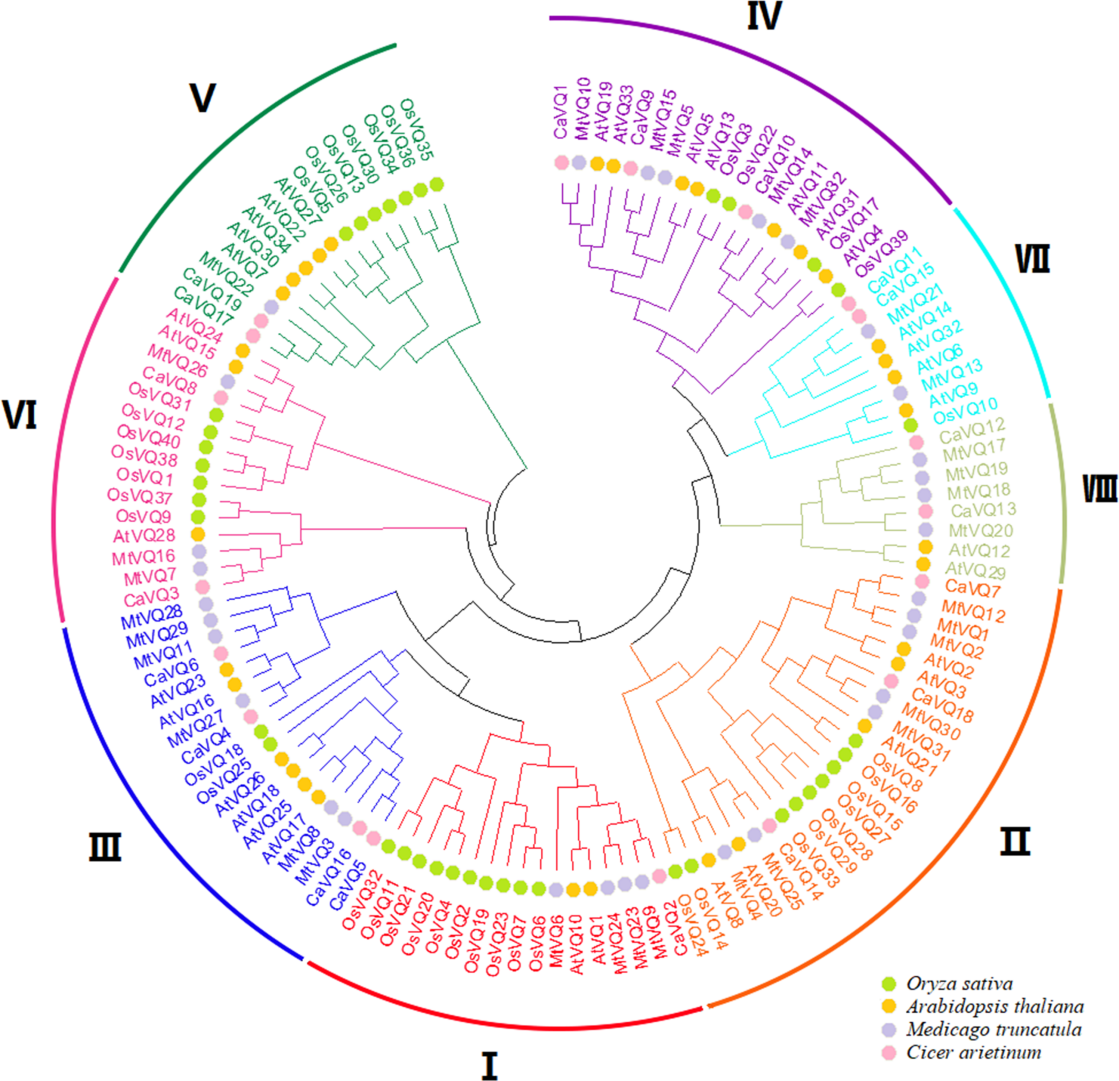
Phylogenetic tree analysis of the *VQ* genes in *Cicer arietinum*, *Medicago truncatula*, *Glycine max*, *Phaseolus vulgaris*, *Arabidopsis thaliana* and *Oryza sativa*. The clusters were designated as group I–VII and indicated in a specific color. Different colored circles represent different species.

We constructed a NJ phylogenetic tree to explore the evolutionary relationship between *VQ* genes in *C. arietinum*, *M. truncatula*, *A. thaliana* and *O. Sativa* (Fig. 1)*.* As shown in Fig. 1, VQ proteins were classified into eight groups*.* Groups II and III contained 10 proteins, respectively. While Group V only contained 3 VQ proteins. We also found that CaVQs and MtVQs were clustered together, suggesting that they might have originated from a common ancestor.

### Conservative motifs and structural analysis

**Figure 2:**
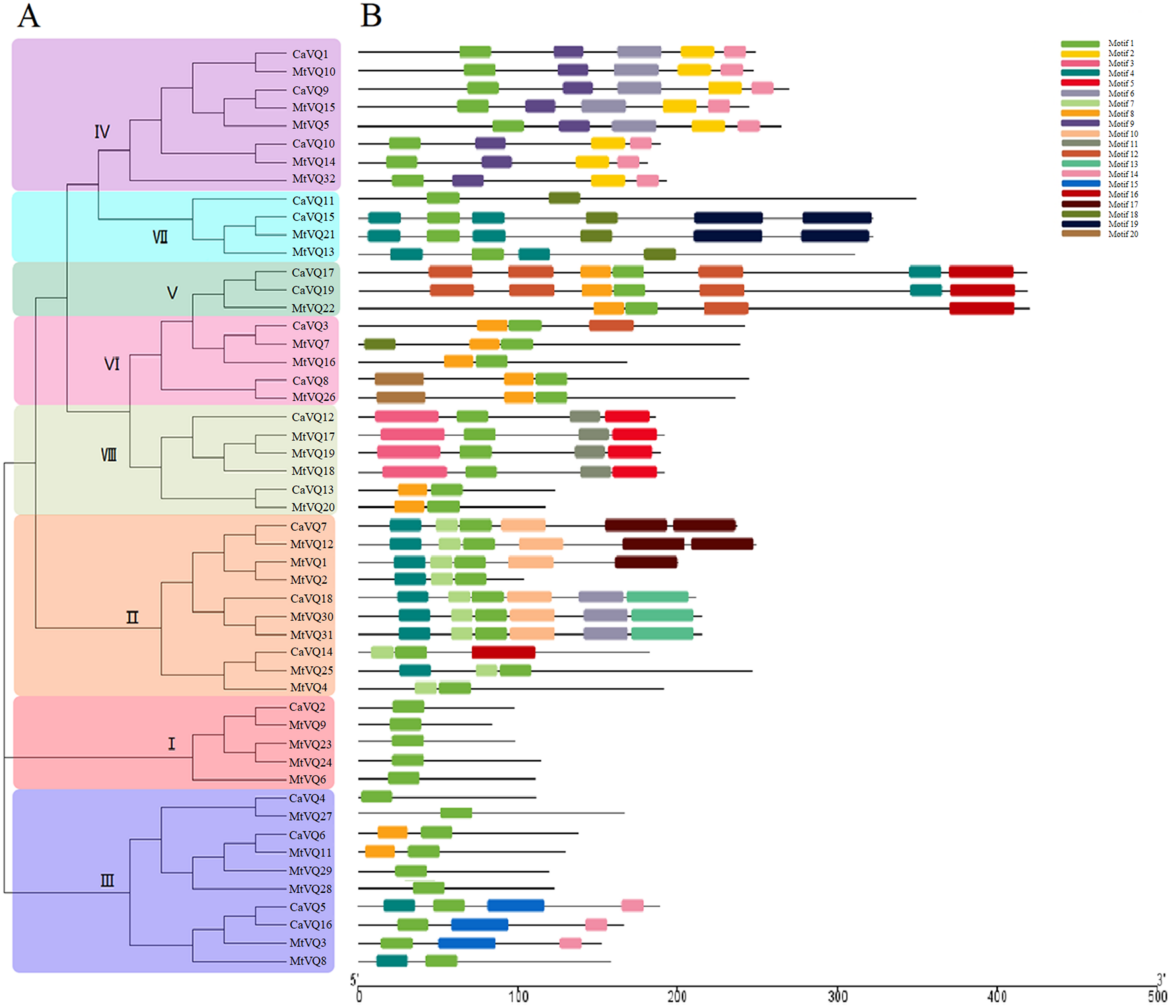
Phylogenetic tree, conserved motifs and gene structure in CaVQs and MtVQs. (A) Phylogenetic relationships (B) Conserved motifs of the VQ proteins. Each motif is represented by a number in colored box.

To analyse the sequence characteristics of the CaVQ and MtVQ proteins, we used the MEME tool to predict their conserved motifs (Fig. 2). A total of 20 motifs describing details of CaVQ and MtVQ proteins were predicted and termed motifs 1–20 (Fig. 2B, Fig. S1). Motif 1 contained the *VQ* domain, which is an essential motif in these proteins. In addition, the VQ proteins in the same group possessed the same conserved motifs, which supported the results of the phylogenetic analysis. For instance, motifs 12 and 16 were especially prominent in Group V, motifs 2 and 9 were observed in Group IV, while motifs 3, 6, and 15 were present only in Groups VIII, IV and III, respectively. Multiple sequence alignment was constructed based on the types of VQ domain proteins (Fig. S2). In this study, four types of VQ motifs, including FxxxVQxLTG (39/51), FxxxVQxFTG (6/51), FxxxVQxLTC (4/51), FxxxVQxVTG (2/57) were identified in CaVQ and MtVQ proteins.

**Table 1:** List of all *VQ* genes identified in the *Cicer arietinum* and *Medicago truncatula*.

Gene name	Gene locus	Chromosome location	Length (aa)	pI	Molecular weight (Da)	Family group
CaVQ1	Ca_00955	chr1:10979073-10980807	249	9.1	27,619.92	IV
CaVQ2	Ca_01025	chr1:12158855-12159148	97	5.75	11,039.64	I
CaVQ3	Ca_01680	chr1:26160930-26161682	242	6.58	26,983.87	VI
CaVQ4	Ca_06894	chr3:31483218-31483553	111	4.06	12,336.44	III
CaVQ5	Ca_11582	chr4:49509075-49509676	189	8.73	21,146.98	III
CaVQ6	Ca_12232	chr5:6215916-6216696	137	5.3	15,752.72	III
CaVQ7	Ca_13983	chr5:33457054-33457908	237	9	26,258.8	II
CaVQ8	Ca_15228	chr6:509649-510383	244	5.92	25,965.88	VI
CaVQ9	Ca_16724	chr6:17665023-17665832	269	9.84	29,261.45	IV
CaVQ10	Ca_17424	chr6:26291509-26292115	189	7.99	21,123.94	IV
CaVQ11	Ca_20005	chr7:18008062-18009111	349	8.48	38,732.05	VII
CaVQ12	Ca_20836	chr7:31217857-31218417	186	9.02	21,083.95	VIII
CaVQ13	Ca_22668	chr8:5942711-5943082	123	9.37	13,733.34	VIII
CaVQ14	Ca_23038	chr8:10629700-10630248	182	6.64	20,155.55	II
CaVQ15	Ca_23593	chr8:17315333-17318137	322	10.66	35,254.12	VII
CaVQ16	Ca_25289	scaffold02951:10588-11088	166	8.29	18,562.07	III
CaVQ17	Ca_26110	scaffold04287:4604-5860	418	6.54	45,765.16	V
CaVQ18	Ca_28175	scaffold14608:1146-1814	211	5.86	22,796.72	II
CaVQ19	Ca_29935	scaffold24605:2988-4244	418	6.54	45,765.16	V
MtVQ1	Medtr1g028910	chr1:9847443-9848042	199	8.89	22,189.14	II
MtVQ2	Medtr1g028920	chr1:9855883-9856191	102	9.7	11,217.65	II
MtVQ3	Medtr1g054055	chr1:23040618-23041237	151	5.83	16,905.05	III
MtVQ4	Medtr1g110570	chr1:49891664-49892623	190	6.51	21,216.83	II
MtVQ5	Medtr2g013950	chr2:3839731-3840815	264	10.18	28,841.89	IV
MtVQ6	Medtr2g019320	chr2:6269503-6270082	109	5.09	11,918.66	I
MtVQ7	Medtr2g035850	chr2:15214004-15215247	237	8.69	26,603.44	VI
MtVQ8	Medtr2g061720	chr2:26182404-26183236	157	8.73	17,727.09	III
MtVQ9	Medtr2g070550	chr2:29736749-29737159	82	4.95	9250.43	I
MtVQ10	Medtr2g079120	chr2:33154057-33155261	246	9.21	27,339.5	IV
MtVQ11	Medtr3g013980	chr3:3851224-3851618	128	5.09	14,727.48	III
MtVQ12	Medtr3g090350	chr3:41104477-41105804	248	9.3	27,225.67	II
MtVQ13	Medtr3g099400	chr3:45563608-45565108	310	9.54	34,283.62	VII
MtVQ14	Medtr4g009950	chr4:2137715-2138666	180	9.36	20,196.77	IV
MtVQ15	Medtr4g088695	chr4:35302060-35303299	244	10.13	26,801.96	IV
MtVQ16	Medtr4g094698	chr4:38752751-38753724	167	9.26	18,856.41	VI
MtVQ17	Medtr4g097280	chr4:40092375-40093365	191	8.42	21,569.32	VIII
MtVQ18	Medtr4g097350	chr4:40121292-40122380	191	9.22	21,691.77	VIII
MtVQ19	Medtr4g097360	chr4:40124928-40125519	188	8.6	21,311.24	VIII
MtVQ20	Medtr5g015190	chr5:5227392-5228070	116	9.57	13,004.54	VIII
MtVQ21	Medtr5g030570	chr5:12954070-12958319	321	10.68	35,263.31	VII
MtVQ22	Medtr5g063310	chr5:26256801-26258739	419	6.45	44,940.2	V
MtVQ23	Medtr6g041990	chr6:14496963-14497256	97	5.88	11,037.38	I
MtVQ24	Medtr6g042010	chr6:14504568-14504918	113	5.55	12,706.14	I
MtVQ25	Medtr6g084200	chr6:31464916-31465653	245	7.05	26,998.07	II
MtVQ26	Medtr7g088620	chr7:34541954-34543335	235	6.91	25,107.02	VI
MtVQ27	Medtr7g089860	chr7:35219206-35219959	165	4.48	18,179.14	III
MtVQ28	Medtr7g115420	chr7:47688748-47689116	122	8.56	13,953.87	III
MtVQ29	Medtr8g040080	chr8:14917344-14917700	118	7.78	13,701.43	III
MtVQ30	Medtr8g093335	chr8:38970208-38971355	214	5.84	23,180.18	II
MtVQ31	Medtr8g093390	chr8:39001408-39002292	214	5.84	23,180.18	II
MtVQ32	Medtr8g095470	chr8:39955693-39958827	192	9.56	21,094.07	IV

We created exon/intron organizational maps based on the coding sequences of each *CaVQ* and *MtVQ* gene (Fig. S3) and found that only 4 *VQ* genes (*CaVQ1*, *CaVQ15*, *MtVQ21* and *MtVQ32*) had introns. Among them, two *VQ* genes belonged to Group VII. The majority of *VQ* genes in *C. arietinum* and *M. truncatula* lacked introns. Furthermore, Group V members had longer coding regions than the others, and Group I members had shorter coding regions than the others.

### Chromosomal locations and gene duplication

**Figure 3:**
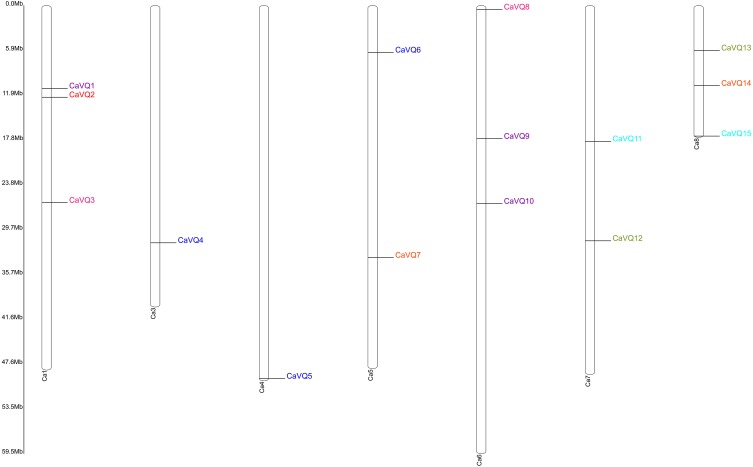
Chromosome location of *CaVQ* genes in *Cicer arietinum.* Genes of different groups are expressed in different colors.

**Figure 4:**
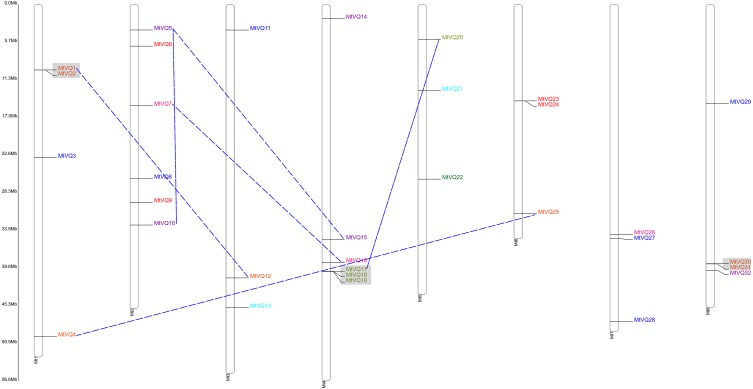
Chromosome location and duplication events analysis of *MtVQ* genes in *Medicago truncatula*. Genes of different groups are expressed in different colors. Gray boxes showed tandem duplication events and blue dashed lines showed fragment duplication events.

The locations of the *VQ* genes revealed that they were unevenly distributed on their corresponding chromosomes (Table 1, Figs. 3 and 4). *VQ* genes were identified in 7 of 8 *C. arietinum* chromosomes and in all *M. truncatula* chromosomes. In *C. arietinum*, four genes (*CaVQ16*, *CaVQ17*, *CaVQ18* and *CaVQ19*) could not be mapped on any chromosome. In *M. truncatula*, there were four gene clusters (*MtVQ1*-*MtVQ2*, *MtVQ17*-*MtVQ18*-*MtVQ19*, *MtVQ23*-*MtVQ24* and *MtVQ30*-*MtVQ31*) located on chromosomes 1, 4, 6 and 8, respectively.

The gene duplication analysis (Figs. 3 and 4) revealed that there were 4 and 6 gene pairs originating in tandem duplication and segment duplication events in *M. truncatula*; however, there was no gene duplication event in *C. arietinum*. Three gene clusters were formed by tandem duplication located on chromosomes 1, 4, and 8 in *M. truncatula*.

### Synteny analysis of *VQ* genes

**Figure 5:**
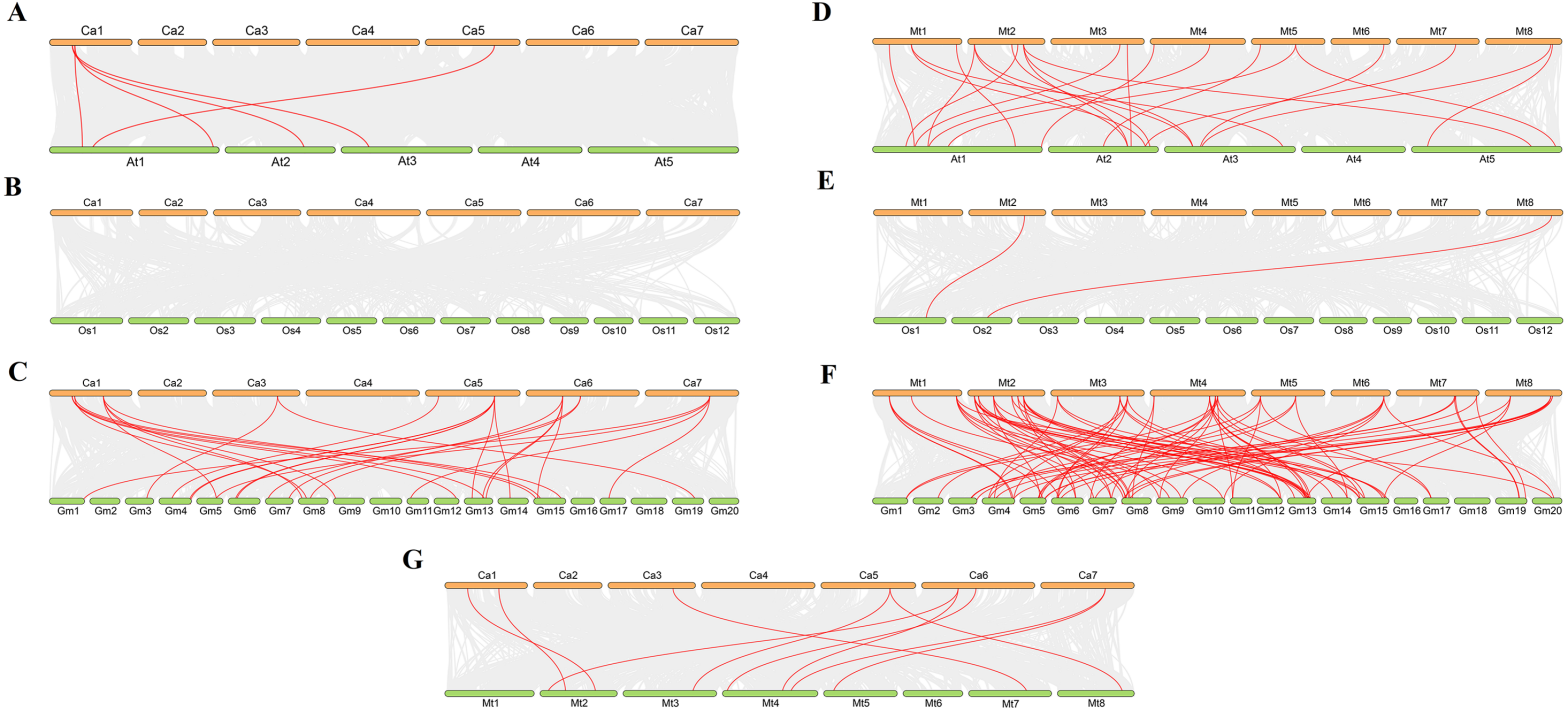
Synteny analysis of VQ genes between *Cicer arietinum*, *Medicago truncatula* and plant species. Synteny analysis of the VQ genes between (A) *Cicer arietinum* and *Arabidopsis thaliana*; (B) *Cicer arietinum* and *Oryza sativa*; (C) *Cicer arietinum* and *Glycine max*; (D) *Medicago truncatula* and *Arabidopsis thaliana*; (E) *Medicago truncatula* and *Oryza sativa*; (F) *Medicago truncatula* and *Glycine max*; (G) *Cicer arietinum* and *Medicago truncatula*. Gray lines in the background indicate the collinear blocks within the *Cicer arietinum* or *Medicago truncatula* and other plant genomes, while the red lines highlight the syntenic VQ gene pairs.

We analysed collinearity diagrams between the *VQ* genes in *C. arietinum*, *M. truncatula*, and other model plants, such as *A. thaliana*, *O. sativa* and *G. max* (Fig. 5). We found that the *VQ* genes in *C. arietinum* had the most homologous gene pairs with *VQ* genes in *G. max* (37), followed by *A. thaliana* (8) (Figs. 5A–5C). Similarly, the *VQ* genes in *M. truncatula* had the most homologous gene pairs with *VQ* genes in *G. max* (87), followed by *A. thaliana* (23) and *O. sativa* (2) (Figs. 5D–5E). However, no homologous gene pairs were observed between *C. arietinum* and *O. sativa* (Fig. 5F). Ten homologous gene pairs were observed between the *CaVQ* and *MtVQ* genes (Fig. 5G). In addition, one *VQ* gene in *C. arietinum* and *M. truncatula* matched more than one *VQ* gene in other plants.

### *Ka/Ks* of *VQ* genes

To better understand the selection pressure acting on paralogous *MtVQ* gene pairs and orthologous *VQ* gene pairs between *C. arietinum* and *M. truncatula*, we calculated their *Ka/Ks* substitution ratios (Table 2). Our results suggest that the *Ka/Ks* values of most gene pairs were <1; the *Ka/Ks* value of only one gene pair (*MtVQ19/MtVQ17*) was >1, indicating that they had primarily evolved under purifying selection. We also found that the differentiation time of *VQ* genes in *C. arietinum* and *M. truncatula* was between 110 and 190 MYA and that the differentiation time of paralogous *VQ* gene pairs in *M. truncatula* was primarily between 3 and 11 MYA.

**Table 2:** Ka, Ks and Ka/Ks values calculated for homologous *VQ* gene pairs.

Gene 1	Gene 2	Ka	Ks	Ka/Ks	Differentiation time
MtVQ8	MtVQ3	0.506	3.245	0.156	265.963
MtVQ15	MtVQ5	0.271	0.815	0.333	66.820
MtVQ17	MtVQ18	0.072	0.109	0.658	8.933
MtVQ18	MtVQ19	0.108	0.134	0.808	10.982
MtVQ19	MtVQ17	0.053	0.045	1.198	3.651
MtVQ24	MtVQ23	0.198	0.275	0.721	22.517
MtVQ3	CaVQ5	0.915	1.380	0.664	113.082
MtVQ5	CaVQ9	1.278	2.104	0.607	172.465
MtVQ7	CaVQ3	1.099	2.227	0.493	182.560
MtVQ9	CaVQ2	0.960	2.165	0.443	177.493
MtVQ10	CaVQ1	1.585	2.299	0.689	188.451
MtVQ11	CaVQ6	0.900	1.379	0.653	113.072
MtVQ12	CaVQ7	0.939	1.607	0.584	131.760
MtVQ15	CaVQ9	1.368	1.706	0.802	139.867
MtVQ25	CaVQ14	1.289	2.504	0.515	205.213
MtVQ26	CaVQ8	1.132	1.837	0.616	150.581
MtVQ27	CaVQ4	1.314	2.717	0.484	222.709

### *Cis*-element analysis of *VQ* genes

To investigate gene function and regulation, we analyzed cis-elements in the promoters of *CaVQs* and *MtVQs* (Table S2, Fig. S4). The multiple light responsive elements were observed in the *VQ* genes (e.g., G-Box, GT1-motif, 3-AF1 binding site and TCT-motif) (Table S2). Furthermore, some *cis*-elements participated in plant growth and development (e.g., circadian, RY-element, and CAT-box) (Fig. S4). Other *cis*-elements could be classified into two major groups: hormone responsive and abotic stress. Ten *cis*-elements are involved in hormone responses, including ABRE, P-box, TATC-box and AuxRR-core, and five *cis*-elements are related to stress, i.e., ARE, LTR, MBS, TC-rich repeats and GC-motif. In addition, we found that several *VQ* genes contained W-box motifs, which are binding sites for WRKY transcription factors (Wang et al., 2010a).

### In silico analysis of *VQ* genes in different tissues

**Figure 6:**
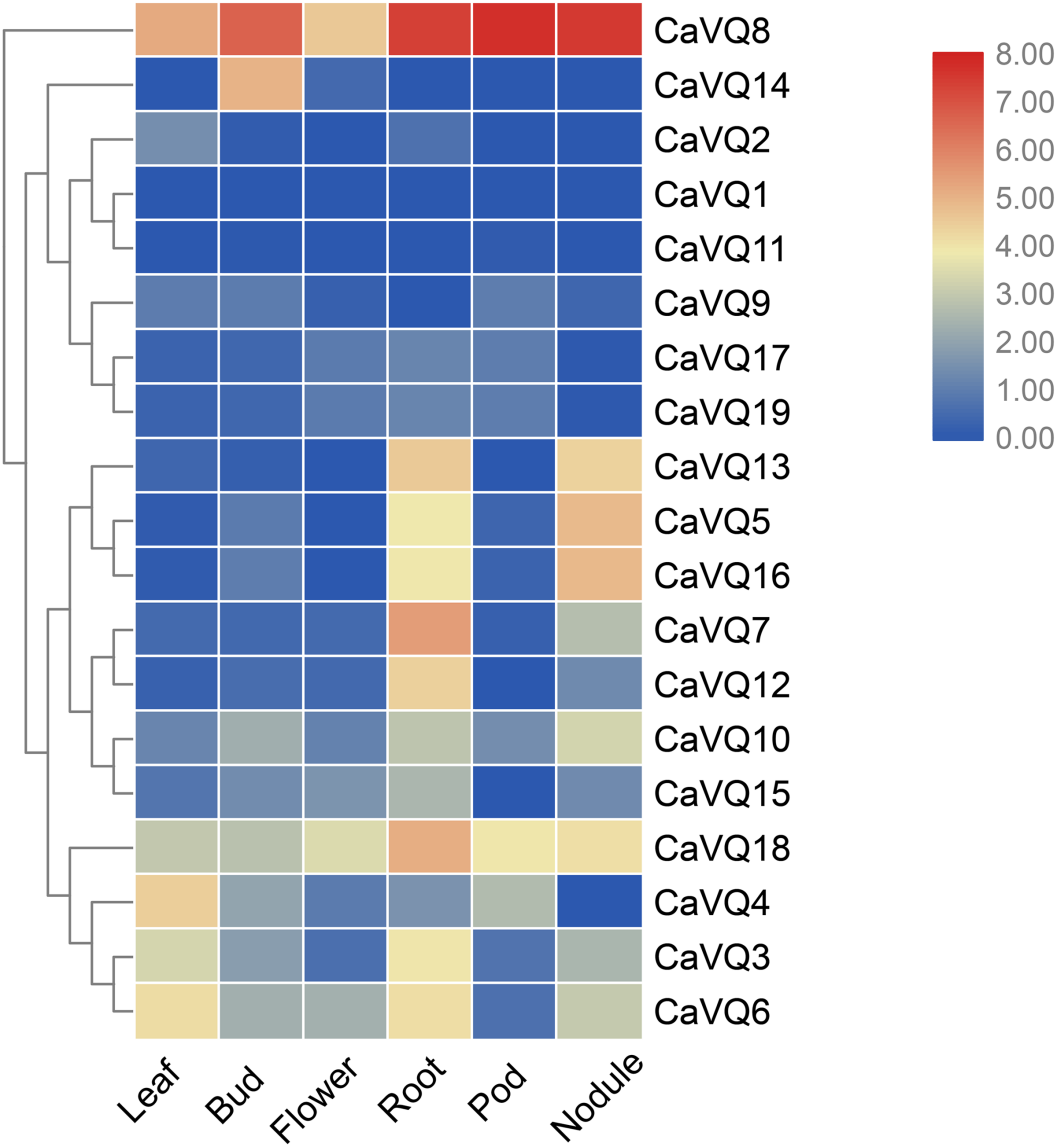
Expression analysis of *CaVQ* genes in different tissues. The gene expression values are square-root transformed fragments per kilo-bases per million mapped reads (FPKM). Different colors in map represent gene transcript abundance values as shown in the color bar.

**Figure 7:**
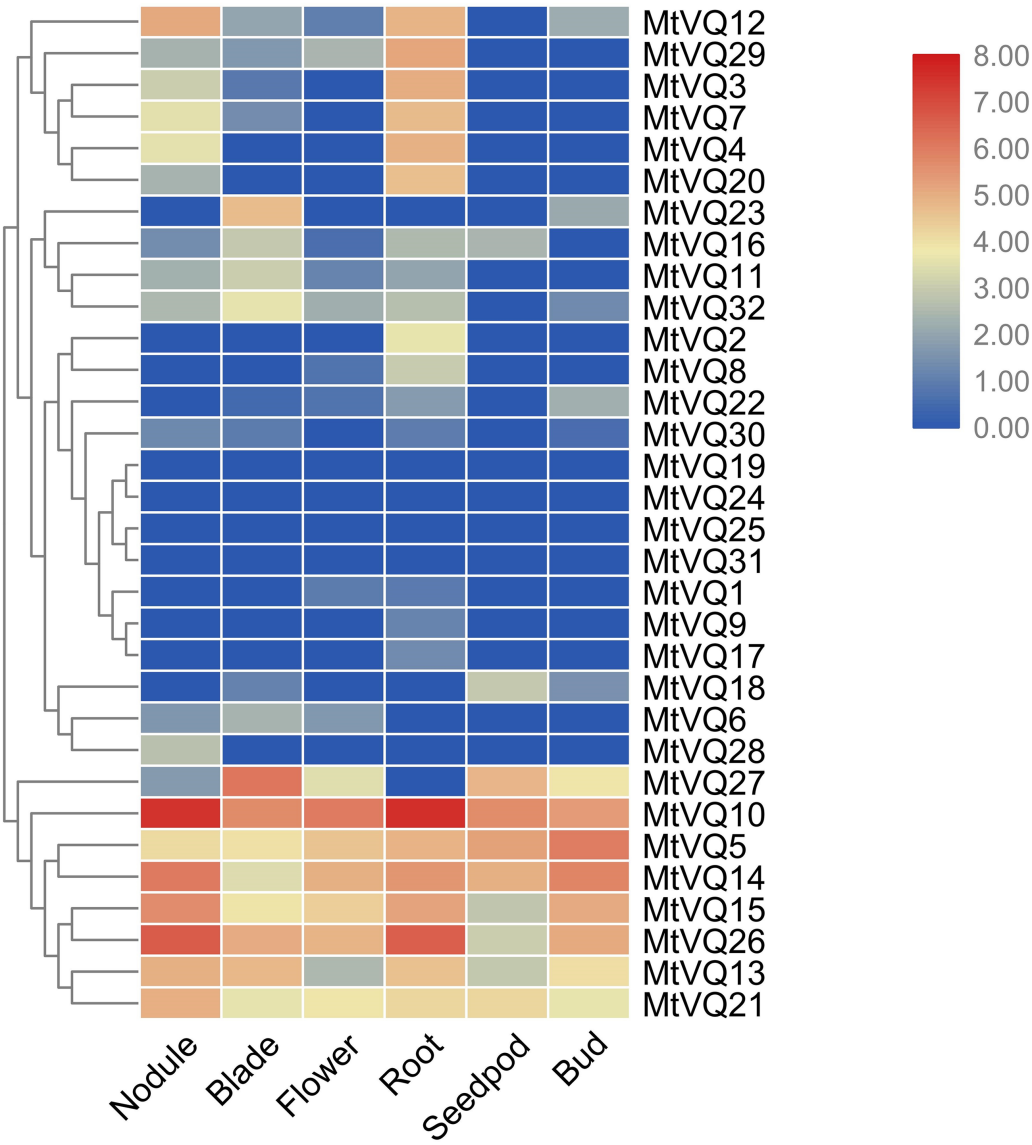
Expression analysis of *MtVQ* genes in different tissues. The gene expression values are square-root transformed fragments per kilo-bases per million mapped reads (FPKM). Different colors in map represent gene transcript abundance values as shown in the color bar.

We investigated the expression profiles of *CaVQ* and *MtVQ* genes in various tissues using high-throughput sequencing data from NCBI, including leaf, bud, flower, root, pod, nodule in *C. arietinum* and nodule, blade, flower, root, seedpod, bud in *M. truncatula*. As shown in Fig. 6, most *CaVQ* genes exhibited tissue-specific expression patterns. Seven *CaVQ* genes (*CaVQ13, 5, 16, 7, 12, 10* and *15*) were highly expressed in the root and nodule; four *CaVQ* genes (*CaVQ18, 4* and *6*) were highly expressed in leaf and root; *CaVQ14* was expressed only in the bud; *CaVQ8* was highly expressed in the six tissues; and six *CaVQ* genes (*CaVQ2, 1, 11, 9, 17* and *19*) were expressed at low levels in all tissues. Most *CaVQ* genes exhibited tissue-specific expression patterns. In *M. truncatula* (Fig. 7), six genes (*MtVQ12, 29, 3, 7, 4* and *20*) were highly expressed in the nodule and root; four *MtVQ* genes (*MtVQ23, 16, 11* and *32*) were expressed only in the blade; eight *MtVQ* genes (*MtVQ27, 10, 5, 14, 15, 26, 13* and *21*) were highly expressed in all detected tissues; and the other *MtVQ* genes were expressed at low levels in six tissues.

### Expression patterns of *VQ* genes under abiotic stresses

**Figure 8:**
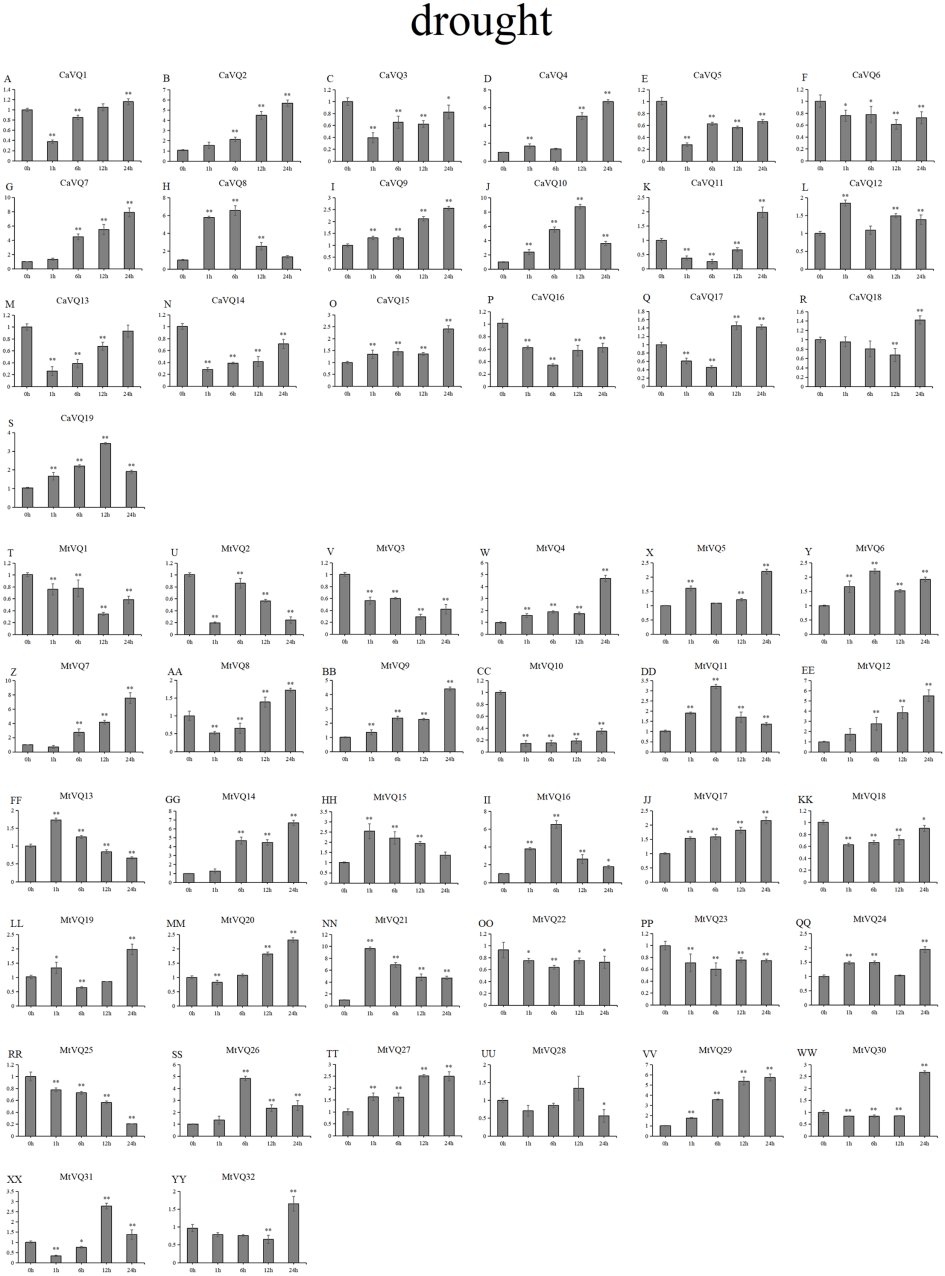
qRT-PCR validation of *VQ* genes in the response to drought treatment. Stress treatments and time course are described in “Materials & Methods”. (A-YY) represent different genes which were used in qRT-PCR analysis. Asterisks on top of the bars indicating statistically significant differences between the stress and counterpart controls (^∗^*p* < 0.05, ^∗∗^*p* < 0.01).

**Figure 9:**
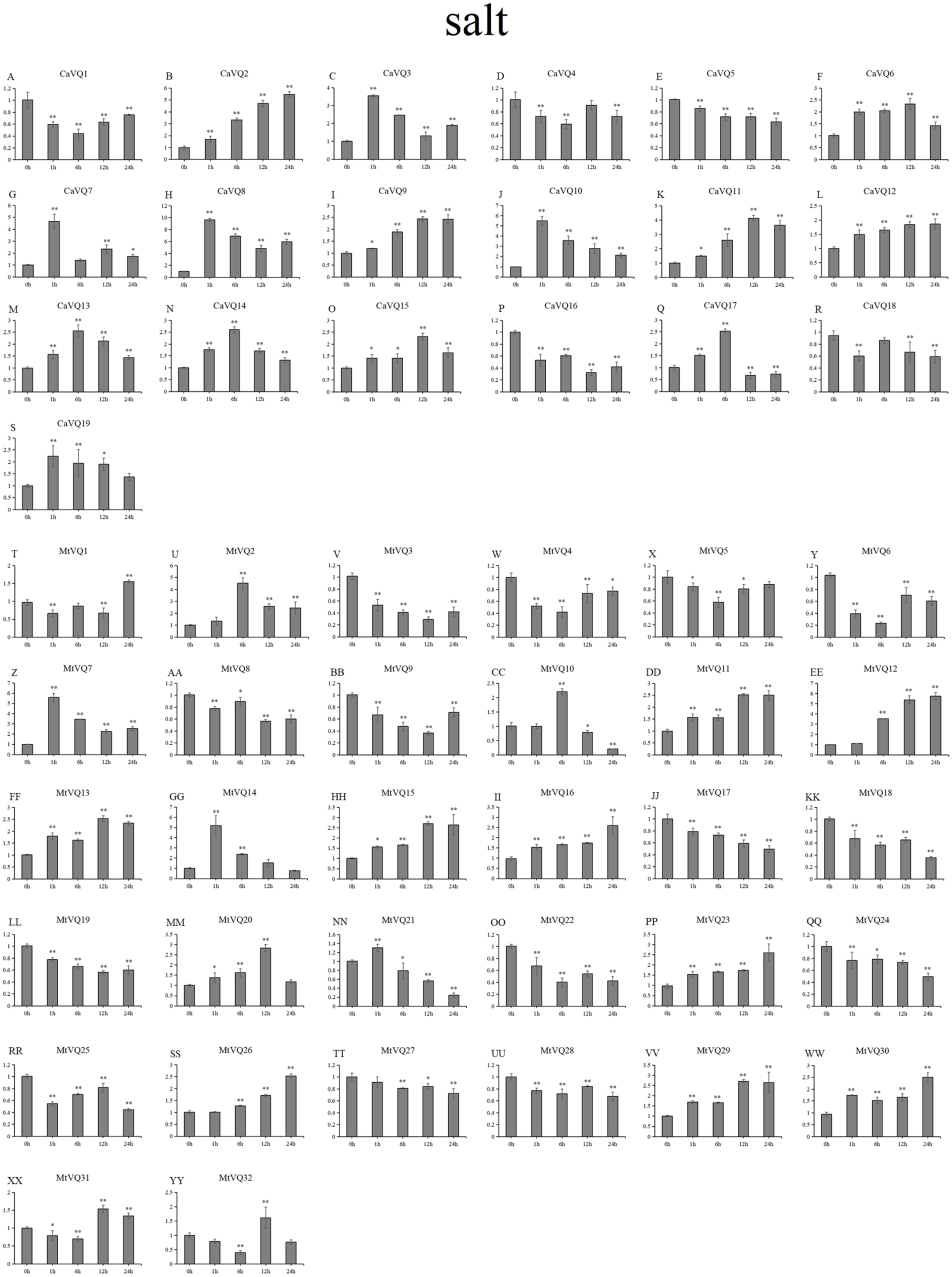
qRT-PCR validation of *VQ* genes in the response to salt treatment. Stress treatments and time course are described in “Materials & Methods”. (A-YY) represent different genes which were used in qRT-PCR analysis. Asterisks on top of the bars indicating statistically significant differences between the stress and counterpart controls (^∗^*p* < 0.05, ^∗∗^*p* < 0.01).

**Figure 10:**
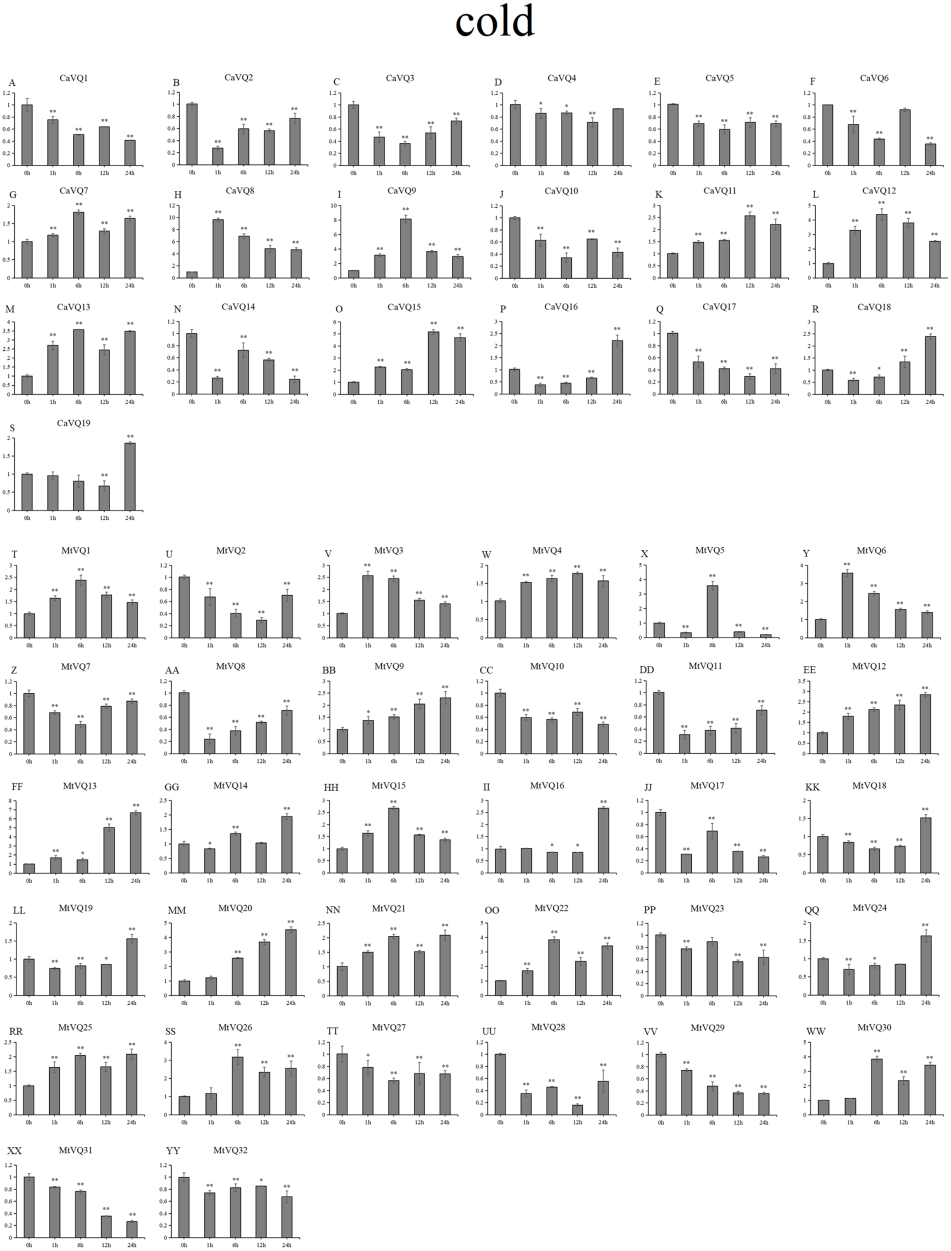
qRT-PCR validation of *VQ* genes in the response to cold treatment. Stress treatments and time course are described in “Materials & Methods”. (A-YY) represent different genes which were used in qRT-PCR analysis. Asterisks on top of the bars indicating statistically significant differences between the stress and counterpart controls (^∗^*p* < 0.05, ^∗∗^*p* < 0.01).

**Figure 11:**
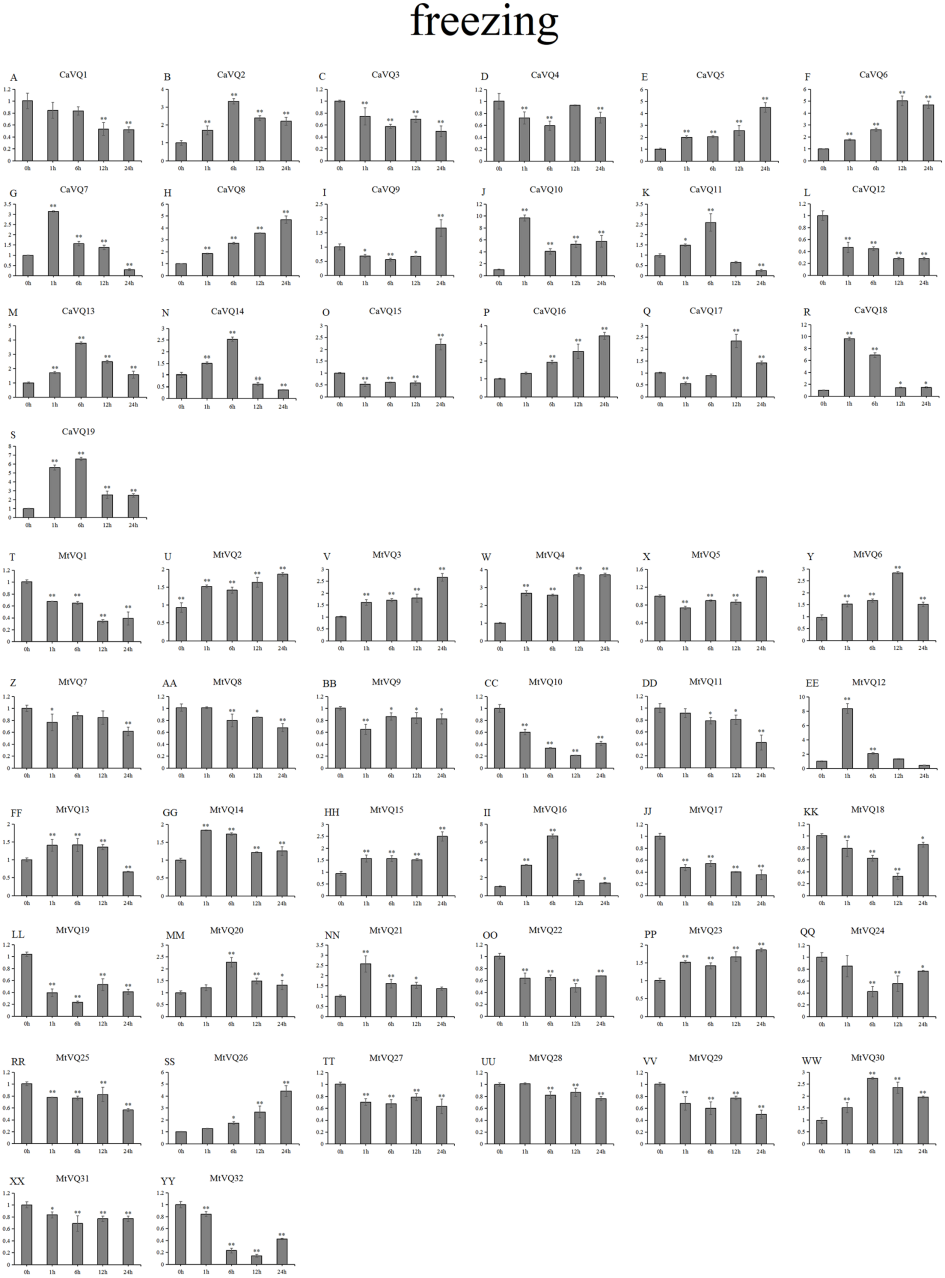
qRT-PCR validation of *VQ.* genes in the response to freezing treatment. Stress treatments and time course are described in “Materials & Methods”. (A-YY) represent different genes which were used in qRT-PCR analysis. Asterisks on top of the bars indicating statistically significant differences between the stress and counterpart controls (^∗^*p* < 0.05, ^∗∗^*p* < 0.01).

In our study, we examined the expression patterns of *CaVQ* and *MtVQ* genes under different stress conditions to identify which genes might take part in abiotic stress responses. We performed a qRT-PCR to analyse the expression profiles of the *MtVQ* and *CaVQ* genes under different stresses, such as drought, salt, cold and freezing (Figs. 8–11).

After drought treatment (Fig. 8), the expression of five *CaVQ* genes (*CaVQ2*, *4*, *7*, *9*, and *15*) and eight *MtVQ* genes (*MtVQ4, 5, 9, 12, 14, 17, 27,* and *29*) significantly increased (more than 1.5-fold) and peaked at 24 h. In addition, six *CaVQ* genes (*CaVQ1, 5, 11, 13, 14* and *17*) and six *MtVQ* (*MtVQ2, 3, 8, 18, 20* and *31*) genes were significantly downregulated at early time points, then their expression levels increased. Three genes (*MtVQ6*, *16* and *26*) were upregulated (more than 2-fold) and peak at 6 h. Except these, *MtVQ10* was significantly downregulated more than 1.5-fold during the drought stress. During salt stress (Fig. 9), eight *CaVQ* genes (*CaVQ3, 7, 8, 10, 13, 14, 17* and *19*) and four *MtVQ* genes (*MtVQ7, 10, 14* and *21*) were significantly induced at early time points. On the contrary, four *CaVQ* genes (*CaVQ1, 4, 16* and *18*) and three *MtVQ* genes (*MtVQ1, 3* and *6*) were downregulated at 1 h and their expression levels were further decreased at 6 h with the exception *CaVQ16*, *CaVQ18* and *MtVQ1*. For cold treatment (Fig. 10), seven *CaVQ* genes (*CaVQ7, 8, 9, 11,12, 13* and *15*) and eleven *MtVQ* genes (*MtVQ1, 4, 6, 9, 12, 13, 15, 20, 21, 22* and *25*) were rapidly upregulated at early time points. Among these genes, four *CaVQ* genes (*CaVQ7, 9, 12* and *13*) and three *MtVQ* genes (*MtVQ1, 15* and *22*) showed the highest expression levels at 6 h. In contrast, seven *CaVQ* genes (*CaVQ1, 2, 3, 5, 10, 14* and *17*) and twelve *MtVQ* gene (*MtVQ2, 7, 8, 10, 11, 17, 23, 27, 28, 29, 31* and *32*) showed a trend of downregulation from 1 h to 24 h. Interestingly, *MtVQ5* was upregulated about 2-fold at 6 h but downregulated more than 4-fold at 24 h. For the case of freezing treatment (Fig. 11), six *CaVQ* genes (*CaVQ2, 7, 13, 14, 18* and *19*) and five *MtVQ* genes (*MtVQ12, 16, 20, 21* and *30*) at early time points (1 h or 6h) exhibited at least a 1.5-fold increase in expression compared to the untreated control. On the contrary, four *CaVQ* genes (*CaVQ1, 3, 4* and *12*) and thirteen *MtVQ* genes (*MtVQ1, 10, 11, 17, 18, 19, 22, 24, 25, 27, 29, 31* and *32*). Interestingly, *MtVQ12* was rapidly upregulated at 1 h (more than 7-fold), then the expression level decreased.

To explore the stress-specific distribution of the *VQ* gene family under four abiotic stresses (drought, salt, cold and freezing), we compared the gene expression similarly between *C. arietinum* and *M. truncatula* under a combination of all stresses, and the results are shown in Fig. S5. Some *VQ* genes were exclusively induced, and certain *VQ* genes were exclusively inhibited. Under four stresses, three *CaVQ* genes (*CaVQ2, 7* and *8*) were upregulated at all the time points; only *CaVQ17* was downregulated at early time point but no gene was downregulated at late time point. six *MtVQ* genes (*MtVQ12, 13, 14, 15, 21* and *26*) were upregulated and six *MtVQ* genes (*MtVQ8, 18, 19, 28, 31* and *32*) were downregulated genes under all four stresses at early time points. At the same time, five *MtVQ* genes (*MtVQ15,16,20,21* and *23*) were exclusively induced and two *MtVQ* genes (*MtVQ21* and *28*) were repressed under all stresses at later time points.

### Expression patterns of homologous genes under abiotic stresses

We found that most homologous genes between *MtVQ* and *CaVQ* genes showed the same expression patterns under abiotic stresses (Fig. S6). Under drought stress, five gene pairs (*CaVQ1*/*MtVQ10*, *CaVQ7*/*MtVQ12*, *CaVQ9*/*MtVQ5*, *CaVQ12*/*MtVQ17* and *CaVQ13*/*MtVQ20*) showed similar expression patterns. Under salt stress, only three gene pairs (*CaVQ3*/*MtVQ7*, *CaVQ10*/*MtVQ14* and *CaVQ13*/*MtVQ20*) showed similar expression patterns in *C. arietinum* and *M. truncatula*, while other gene pairs (*CaVQ1/MtVQ10*, *CaVQ7/MtVQ12*, *CaVQ9/MtVQ5* and *CaVQ12/MtVQ17*) exhibited opposing expression patterns. The expression levels of most *VQ* gene pairs were increased at early time points during salt stress. Under cold stress, five gene pairs (*CaVQ1*/*MtVQ10*, *CaVQ3*/*MtVQ7*, *CaVQ7*/*MtVQ12*, *CaVQ9*/*MtVQ5* and *CaVQ13*/*MtVQ20*) had the same expression pattern; however, *CaVQ10*/*MtVQ14* and *CaVQ12*/*MtVQ17* showed opposite expression patterns. Similarly, under freezing stress, most gene pairs showed the same expression patterns, among them, three gene pairs (*CaVQ7*/*MtVQ12*, *CaVQ10*/*MtVQ14 and CaVQ13*/*MtVQ20*) exhibited a trend of initially rising and then falling.

## Discussion

VQ proteins are plant-specific proteins involved in the regulation of plant growth, development and responses to various environmental stresses in plants (Chu et al., 2016; Guo et al., 2018; Zhong et al., 2018; Cao et al., 2018). *VQ* genes have been identified in various plants, such as *A. thaliana*, *O. sativa*, *Z. mays*, *G. max* and *V. vinifera* (Kim et al., 2013a; Cheng et al., 2012; Zhou et al., 2016; Wang et al., 2015). Legumes, such as *C. arietinum* and *M. truncatula*, are widely cultivated and have high nutritional and economic value (Kim et al., 2013b; Cheng et al., 2012; Zhou et al., 2016; Wang et al., 2015). However, systematic analyses of *VQ* genes in *C. arietinum* and *M. truncatula* are lacking. Herein, a total of 19 and 32 *VQ* genes were identified in *C. arietinum* and *M. truncatula*, respectively. Although the genome size of *C. arietinum* is twice larger than that of *M. truncatula*, the number of *VQ* genes in *M. truncatula* is much larger than that in *C. arietinum*, indicating that a large number of *CaVQ* genes have been lost during evolution (Wang et al., 2017a). We systematically analysed the structural and functional characteristics of the *CaVQ* genes and *MtVQ* genes to explore their evolutionary relationships and provide a theoretical basis for further research.

*CaVQ* genes and *MtVQ* genes were closely related, based on the phylogenetic analyse, we found that the CaVQs and MtVQs were always clustered together. The gene structure analysis suggested that 94.74% (18/19 genes) of *CaVQ* genes and 90.63% (29/32 genes) of *MtVQ* genes did not contain introns. These results are consistent with the previous studies that reported the *VQ* genes in *Z. mays* (54, 88.5%) (Song et al., 2016) , *O. sativa* (37, 92.5%) (Kim et al., 2013a), and *A. thaliana* (30, 88.2%) (Cheng et al., 2012) without introns. While, a smaller number of moss *VQ* motif-containing genes (7/25, 28%) are not possess introns (*Jing & Lin et al., 2015*). Comparative these plants (higher plants *C. arietinum*, *M. truncatula*, *A. thaliana*, *Z. mays*, *O. sativa*, and lower plants, moss) indicate that most *VQ* genes have lost introns during the long evolutionary period. Based on the multiple sequence alignment, we found that there are four types in *VQ* domain of CaVQ and MtVQ proteins (LTG, FTG, LTC, VTG), however, there are six types of AtVQ proteins (LTG, LTS, LTD, FTG, VTG, YTG) (Cheng et al., 2012) and four types of OsVQ proteins (ITG, LTG, VTG, FTG) (Kim et al., 2013a) in previous studies. Except these, we found that a unique and conserved sequence “FxxxVQxLTC” in four VQ proteins (MtVQ17-19 and CaVQ12). The conserved motif analysis showed that *CaVQ* genes and *MtVQ* genes were very closely related. Both *CaVQ* genes and *MtVQ* genes showed similar motif patterns in the same groups, such as motif 2 and motif 7 were specifically exist in all members of group IV and II, respectively. These results suggest that *CaVQ* and *MtVQ* genes may originate from a common ancestor.

Segmental and tandem duplication events are major expansion methods in the plant genome (Storz, 2009; Kaltenegger, Leng & Heyl, 2018). In the *MtVQ* gene family, 6 gene pairs originated from segmental duplication and 4 gene pairs were involved in tandem duplication. These results are similar to those found in *Brassica rapa* and pears (Cao et al., 2018; Zhang et al., 2015), suggesting that segmental duplication events are a common expansion mechanism in the VQ gene family. For gene pairs originating from tandem duplication, they all formed gene clusters on *M. truncatula* chromosomes. However, we did not identify gene duplication event in *CaVQ* genes. Furthermore, we noticed that there were a large number of orthologous gene pairs in *CaVQ* and *MtVQ* genes, which is consistent with the results that *C. arietinum* and *M. truncatula* were closely related based on the phylogenetic analysis. The substitution rates of *Ka* and *Ks* are the basis for analysing the selection pressure in gene duplication events (Wang et al., 2010b). We found that the *Ka/Ks* values of most gene pairs were <1, suggested that they had primarily evolved under purifying selection. During evolution, *C. arietinum* and *M. truncatula* common experienced whole genome triplication (*γ* event) at 130 MYA and whole genome duplication (*β*-event) at 59 MYA, and the differentiation time between them was approximately 30–54 MYA (Wang et al., 2017a). However, the differentiation time of the *VQ* gene pairs in *C. arietinum* and *M. truncatula* was approximately 110–190 MYA. These results indicate that the time of gene differentiation is earlier than that of *C. arietinum* and *M. truncatula* differentiation, the *VQ* gene show high intraspecific polymorphism. Except these, the differentiation time of paralogous gene pairs in *M. truncatula* was about 3–25 MYA, which were later than the time of species differentiation (Wang et al., 2017a).

In higher plants, the *VQ* gene family has critical functions in the process of plant growth, development and response to multiple stresses. In the whole period of pear fruit development, most *PbrVQ* genes are expressed and can play critical roles in pear fruit development (Cao et al., 2018). In bamboo, 11 *VQ* genes are highly express in leaf, early panicle, advanced panicle, root and rhizome tissue, and they are lowly express in shoot (Wang et al., 2017b). In this study, based on silico analysis, the *VQ* genes exhibited tissue-specific expression in both *C. arietinum* and *M. truncatula.* We found that six *MtVQ* genes and four *CaVQ* genes were specifically highly expressed in root and nodule, these results are similar to that in soybean: nine and ten *GmVQ* genes are specifically express in root and nodule, respectively (Wang et al., 2014). We speculate that they may be involve in root and nodule formation and development. There were six *VQ* genes (*CaVQ8*, *CaVQ18*, *MtVQ10*, *MtVQ5*, *MtVQ14*, *MtVQ26*) were highly expressed in flowers, *GmVQ43* and *GmVQ62* affect flowering time of plants, we speculate that these *VQ* genes may involve in regulate flowering time and flower development (Zhou et al., 2016). Some orthologous gene pairs showed similar expression patterns in different tissues. For instance, *CaVQ3*/*MtVQ7* was highly expressed in the nodule and root and *CaVQ8*/*MtVQ26* was highly expressed in all examined tissues. However, the *CaVQ5/MtVQ3* gene pair had different expression patterns, with *CaVQ5* highly expressed in six tissues and *MtVQ3* expressed only in the root. These results suggest that some gene pairs retained similar functions while others produced functional differentiation during the process of evolution (Zhong et al., 2018).

In previous study, the *VQ* gene family is found to be involve in responses to multiple stresses (Cai et al., 2019). Certain *PbrVQ* genes were shown to be highly expressed under GA (Gibberellic acid, GA), salt and black spot disease stresses (Cao et al., 2018). In this study, five *CaVQ* genes (*CaVQ2, 4, 7, 9,* and *15*) and eight *MtVQ* genes (*MtVQ4, 5, 9, 12, 14, 17, 27,* and *29*) significantly upregulated during drought stress, which results are similar to the *OsVQ* genes that twenty-two *OsVQ* genes are upregulated under drought stress (Kim et al., 2013a). Under salt stress, eight *CaVQ* genes (*CaVQ3, 7, 8, 10, 13, 14, 17* and *19*) and four *MtVQ* genes (*MtVQ7, 10, 14* and *21*) were significantly induced. In the *A. thaliana*, similar expression changes among *VQ* genes were also observed, including the expression of *AtVQ9* and *AtVQ15* that changed significantly under salt stress (Cheng et al., 2012). In addition, the VQ genes are also sensitive to temperature changes. Seven *CaVQ* genes (*CaVQ7, 8, 9, 11,12, 13* and *15*) and eleven *MtVQ* genes (*MtVQ1, 4, 6, 9, 12, 13, 15, 20, 21, 22* and *25*) were rapidly upregulated at early time point under cold treatment. Six *CaVQ* genes (*CaVQ2, 7, 13, 14, 18* and *19*) and five *MtVQ* genes (*MtVQ12, 16, 20, 21* and *30*) were upregulated at early time point (1 h or 6h) for the case of freezing treatment. Similar results have been reported in *Chinese cabbage* that *VQ* genes are quickly responsive to heat and cold stresses (Zhang et al., 2015). We speculate that *VQ* genes can respond to various abiotic stresses both in *C. arietinum* and *M. truncatula.* By combining promoter analysis with qRT-PCR analysis, we found that *VQ* genes were responses to multiple abiotic that might be closely related to their promoters. For example, *CaVQ13* and *MtVQ26* which contained *cis*-elements (LTR) involved in low temperature responsiveness, all of them showed upregulated expression both under cold and freezing stresses. Oppositely, four *VQ* genes (*CaVQ3*, *CaVQ4*, *CaVQ8* and *CaVQ9*) contained LTR elements and they were downregulated during cold and freezing stresses. Furthermore, *CaVQ5*, *MtVQ18*, *MtVQ22* and *MtVQ25*, which have TC-rich repeats that can participate in response defence and stress, they were showed downregulated expression patterns in drought and salt stresses. The expression patterns of most orthologous gene pairs are similar while others are different. For instance, three gene pairs (*CaVQ3/MtVQ7, CaVQ10/MtVQ14* and *CaVQ13/MtVQ20*) showed similar expression patterns in *C. arietinum* and *M. truncatula*, while other gene pairs (*CaVQ1/MtVQ10, CaVQ7/MtVQ12, CaVQ9/MtVQ5* and *CaVQ12/MtVQ17*) exhibited opposing expression patterns during the salt stress. These results indicate that the orthologous gene pairs in different plants may undergo functional differentiation in the long-term evolution process that they may have distinct regulatory mechanisms under various abiotic stresses (Jiang, Sevugan & Ramachandran, 2018). Hence, the expression of most *CaVQ* and *MtVQ* orthologous gene pairs have undergone functional divergence, indicating that these gene pairs originate from a common ancestor and they are involve in functional redundancy, while other gene pairs are involve in neo-functionalization or sub-functionalization (Sandve, Rohlfs & Hvidsten, 2018). Taken together, our study suggests that a system analysis of the evolutionary relationship, structure and response to various abiotic stresses may help to elucidate the *CaVQ* and *MtVQ* genes for further functional characterization.

## Conclusions

In conclusion, this study provides the first comprehensive and systematic analysis of the *VQ* gene family in two legumes (*C. arietinum* and *M. truncatula*). A total of 19 and 32 *VQ* genes were identified in *C. arietinum* and *M. truncatula*, respectively. All *VQ* genes fell into eight groups (I-VIII). The *VQ* genes from the same evolutionary branches shared similar motifs and structures, these results suggested that the *VQ* genes of *C. arietinum* and *M. truncatula* might originate from a common ancestor. The selection pressure analysis showed that most homologous pairs were under strong purifying selection by the *VQ* genes. In silico analyses revealed that most *VQ* genes exhibited tissue-specific expression patterns, indicating that they might play crucial roles in different tissues. Finally, qRT-PCR analysis showed that the *VQ* gene family was responsive to abiotic stresses. The results indicating that the *VQ* genes not only participates in regulating plant growth and development, but also responds to abiotic stresses. Our results provide a theoretical basis for the further study of *VQ* gene functions.

##  Supplemental Information

10.7717/peerj.8471/supp-1 Figure S1 Sequence logo of motifs in *CaVQ* and* MtVQ* genes The font size represents the frequency of the respective amino acid.

10.7717/peerj.8471/supp-2 Figure S2 Multiple sequence alignment, gene structure and multiple motifs of CaVQ and MtVQ proteins Amino acids that are conserved throughout are shaded in different colors.

10.7717/peerj.8471/supp-3 Figure S3 Exon/intron structures of *VQ* genes UTR are in green boxes and CDS are in yellow boxes.

10.7717/peerj.8471/supp-4 Figure S4 Number of each *cis*-acting element in the promoter region (1.5 kb upstream of the translation start site) of *CaVQ* and* MtVQ* genes

10.7717/peerj.8471/supp-5 Figure S5 Venn diagram represented stress-specific distribution of CaVQs and MtVQs They were into 4 categories: (A) and (E) early up-regulated, (B) and (F) early down-regulated, (C) and (G) late up-regulated and (D) and (H) late down-regulated, respectively.

10.7717/peerj.8471/supp-6 Figure S6 Expression levels of homologous VQ genes under abiotic stresses A-BB represent the expression levels of different gene pairs.

10.7717/peerj.8471/supp-7 Table S1 List of primers used in qRT-PCR

10.7717/peerj.8471/supp-8 Table S2 List of light responsive elements in the *VQ* genes

## Additional Information and Declarations

### Data Availability

The following information was supplied regarding data availability: The data are available at GenBank: PRJNA413872 and PRJNA80163.
