# Supplementary figures and images for: Genome-wide identification and expression analysis of the VQ gene family in Cicer arietinum and Medicago truncatula
Source: PeerJ. 2020 Feb 4;8:e8471. doi: 10.7717/peerj.8471 (PMC7006518; doi:10.7717/peerj.8471)

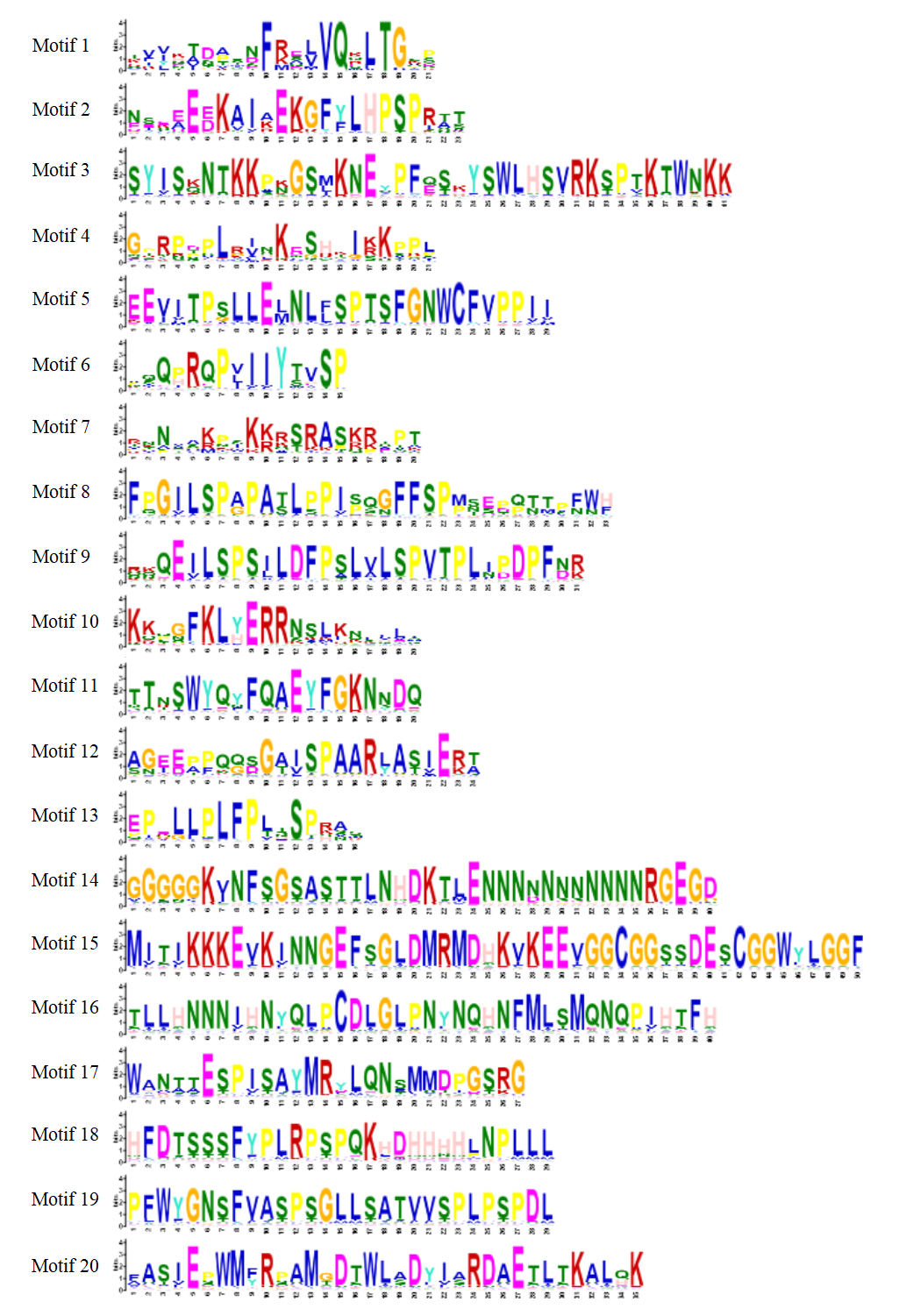

Supplement: Figure S1 — The font size represents the frequency of the respective amino acid. [file peerj-08-8471-s001.png]

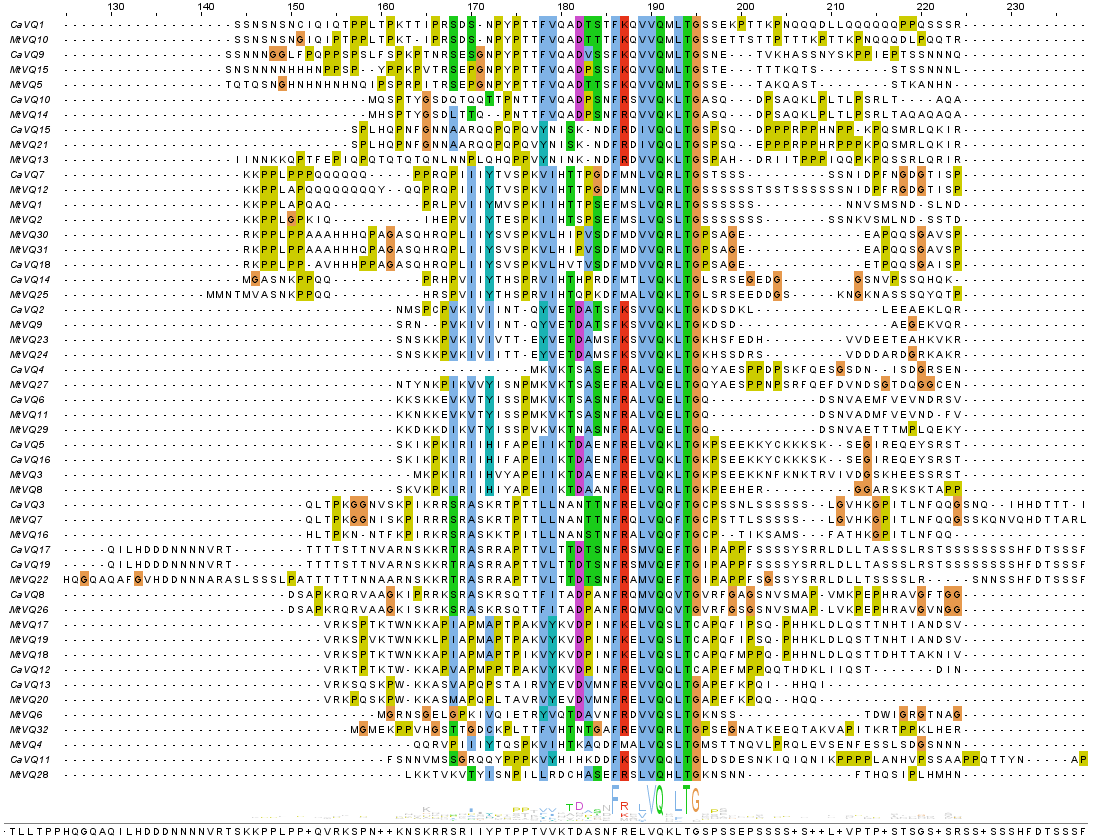

Supplement: Figure S2 — Amino acids that are conserved throughout are shaded in different colors. [file peerj-08-8471-s002.png]

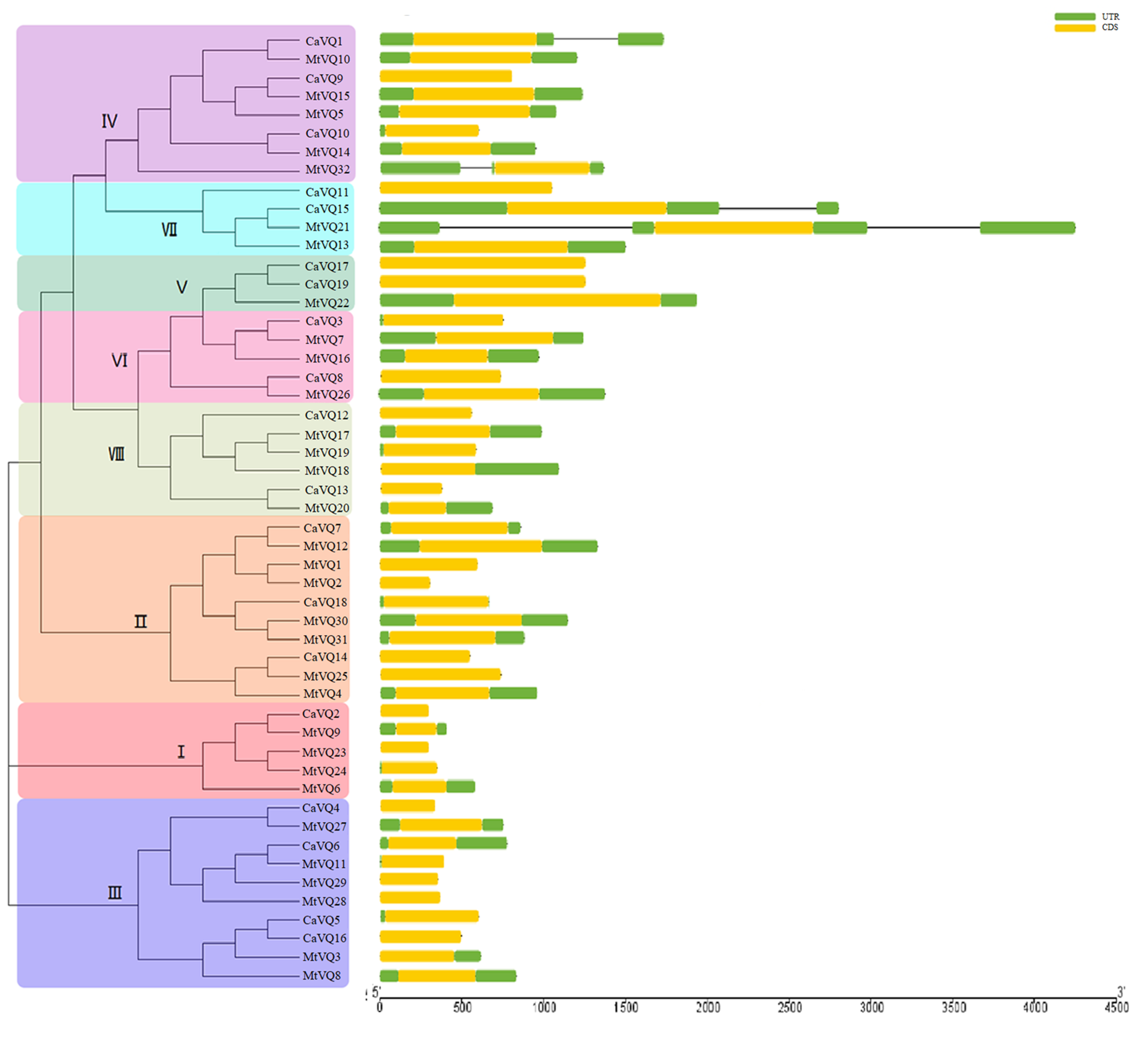

Supplement: Figure S3 — UTR are in green boxes and CDS are in yellow boxes. [file peerj-08-8471-s003.png]

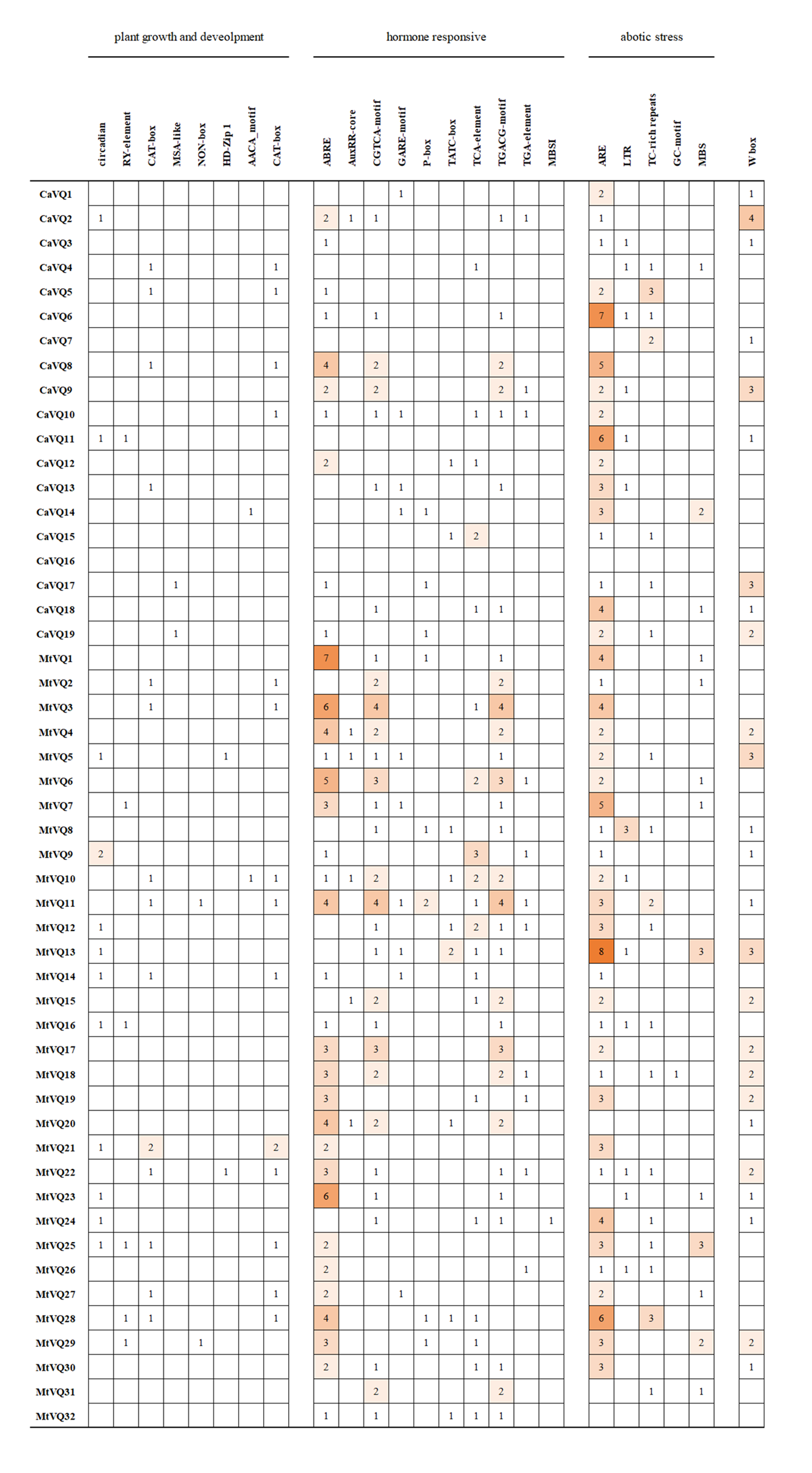

Supplement: Figure S4 [file peerj-08-8471-s004.png]

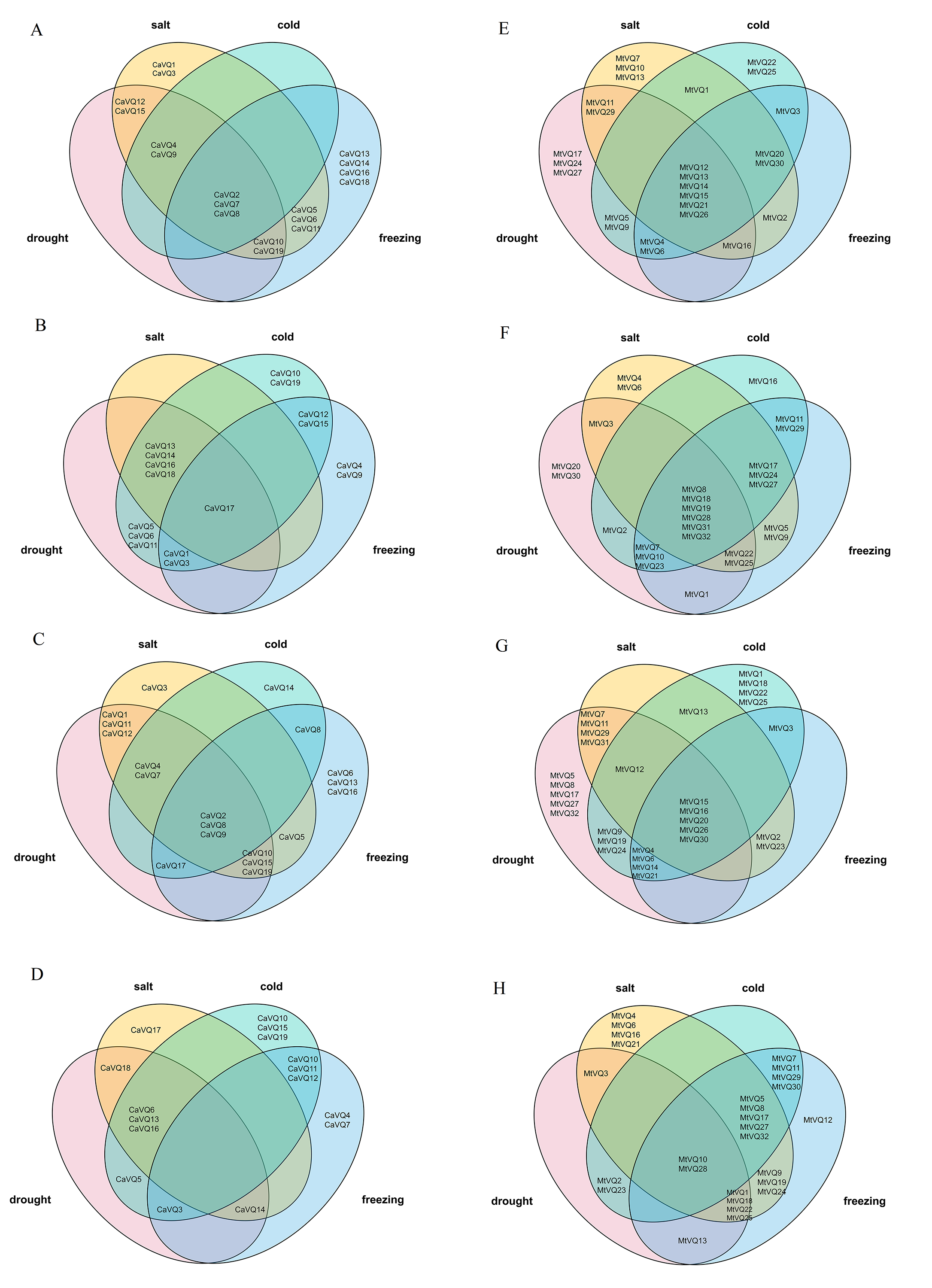

Supplement: Figure S5 — They were into 4 categories: (A) and (E) early up-regulated, (B) and (F) early down-regulated, (C) and (G) late up-regulated and (D) and (H) late down-regulated, respectively. [file peerj-08-8471-s005.png]

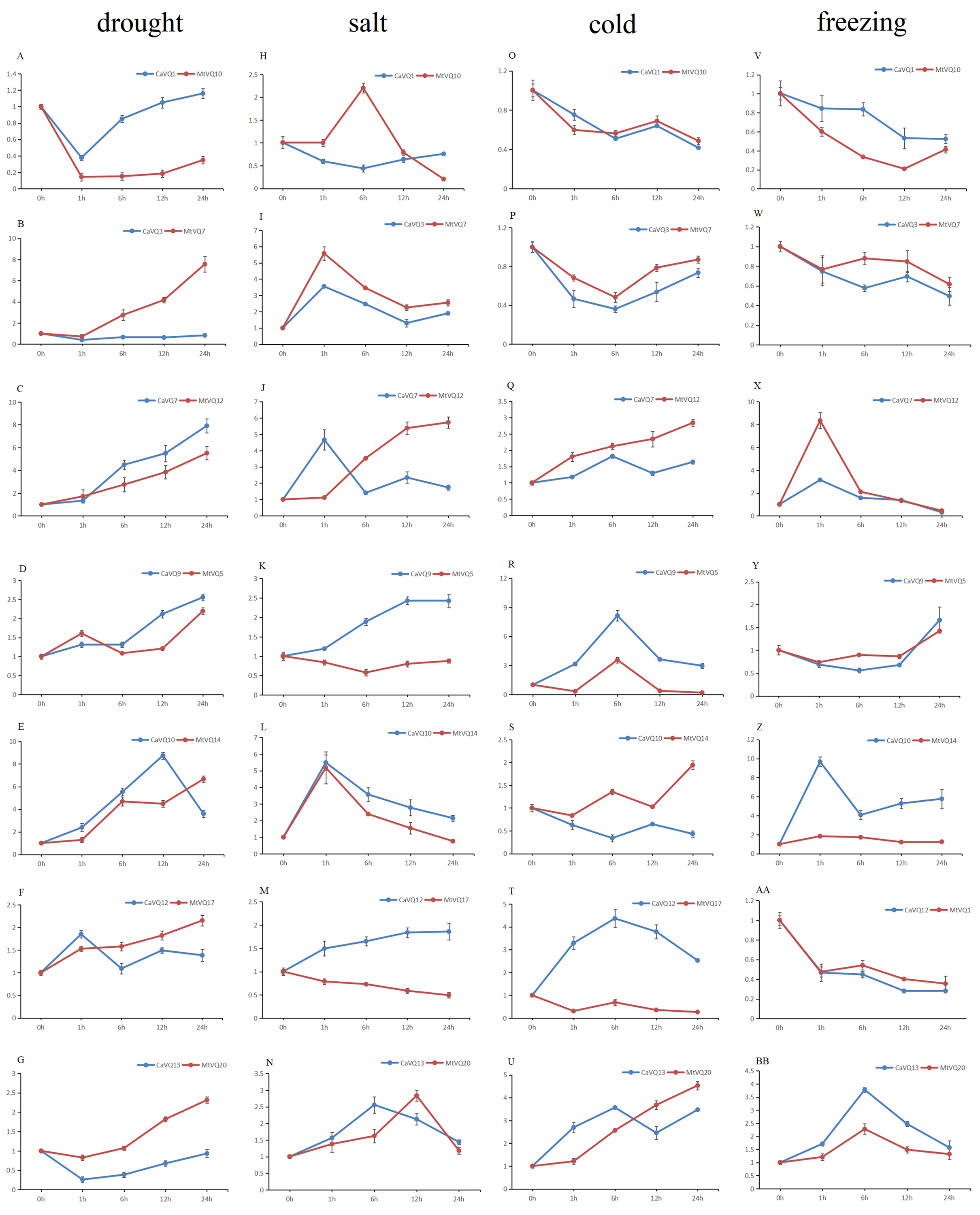

Supplement: Figure S6 — A-BB represent the expression levels of different gene pairs. [file peerj-08-8471-s006.png]
