# Supplementary material for: Genome-wide identification and expression analysis of the VQ gene family in Cicer arietinum and Medicago truncatula
Source: PeerJ. 2020 Feb 4;8:e8471. doi: 10.7717/peerj.8471 (PMC7006518; doi:10.7717/peerj.8471)
Supplement: Table S1 [file peerj-08-8471-s007.docx]

| **Table S1 List of primers used in qRT-PCR** | | |
| --- | --- | --- |
| Gene name | Forward primer (5'-3') | Reverse primer(5'-3') |
| CaVQ1 | TTCCATCACTTGCTCTTAGC | AAGTAACAGGGAAAAGAGGC |
| CaVQ2 | CCTTGTCCTGTGAAGATTGT | GTCGTTTATTGGTGGCATC |
| CaVQ3 | CTCCAAAGGGTGGTAATGTA | GGTTGCTACCTTGTTGAAAG |
| CaVQ4 | ACTGGTCCAAGAACTCACC | CTCACCACTTGGTTTCACTT |
| CaVQ5 | GGGAAACCAAGTGAAGAGA | CACTACTTCCACCACATCCT |
| CaVQ6 | AGCCCTATGAAGGTGAAAAC | TCCTCTTGCATATAGGCTTC |
| CaVQ7 | CATGGAATATTGGAGAGAGG | AGAAGAAATTAGCACCACCC |
| CaVQ8 | ACTCTTGATACGTCAGCGTT | TCACATGACTTTCCACGAC |
| CaVQ9 | ACCTCGGAATAGTCACAATG | GGGTGGTTTTGAGTAGTTTG |
| CaVQ10 | AAACTCCCACTAACTCTCCC | ACAGGTGAAGCCATAACAAC |
| CaVQ11 | CACAACCACAACCTCCTTAT | TCAGTGTCTGACCTTGAACA |
| CaVQ12 | TCACAACCATTGACAGTGAG | GGGCTAATGATAGGAGGAAC |
| CaVQ13 | AAAAGCTTCAGTAGCACCAC | TGTCCTACCACTACTCTCCTG |
| CaVQ14 | ACATTCTCCACGAGTCATTC | TCTGTCGTTTTCCTTGACTC |
| CaVQ15 | GGGTGTTAACAAGTTGGGTA | GTCTAATCTTCTGCAAACGC |
| CaVQ16 | GGGAAACCAAGTGAAGAGA | CACTACTTCCACCACATCCT |
| CaVQ17 | TCACCAAACTACCTCCACTC | TCGAAGCTCTTGTTCTCTTC |
| CaVQ18 | GACGACTTGACGTGGTTACT | GAAAGTAAACCCGAAGGACT |
| CaVQ19 | TCACCAAACTACCTCCACTC | TCGAAGCTCTTGTTCTCTTC |
| MtVQ1 | GAGGACACCAACTACAAGGA | GTGAGACGTTGAACTAGGCT |
| MtVQ2 | GCCCTACACCTCTAACCATA | CGATACCTTATTGGATGACG |
| MtVQ3 | CTTCAAGGAGCACTACAAGC | GAAAACCCACCTAAGTACCC |
| MtVQ4 | GTTCAAAGTCTCACAGGCAT | CACCACCCTTATCACAATTC |
| MtVQ5 | TACACCACTTATACCCGACC | GCCCTAGGAGAAGAAGTAGG |
| MtVQ6 | GACGTTGTTCAAAGCCTTAC | AAGTGGAGGCATCTCTAACA |
| MtVQ7 | GTTCAACATGACACAACAGC | GACAACGAGTTACCCGAAT |
| MtVQ8 | CACACATCTTCTTCAACCTC | CTTTCATGCTCTTCTGGCT |
| MtVQ9 | AAGTGGTGGATGTAGTAGGA | GTGGCATCTCATTAAGAAGC |
| MtVQ10 | ATGAGTAGCAGCAATTCCAA | CTGTTTTGATTGGTGGGATG |
| MtVQ11 | GAGCTCACAGGTCAAGACTC | ATGGCTCCATTGAGGATCT |
| MtVQ12 | GACATGGAAGGGATAGAACA | ACCACTATGAAGTGCAGGAC |
| MtVQ13 | TTTACCACCTCAACCAGAAC | AAGAGGAAGTTGAGGAAACC |
| MtVQ14 | GTTGTTATGGCTTCACCAGT | TGAGATTGAGAGGAAGGAGA |
| MtVQ15 | CTCTTATACCGGACCCTTTT | AGAAGAAGAAGGACCCAAAC |
| MtVQ16 | CTCTTCTCAATGCCAATAGC | CCATAATCAGAGACCTCCAA |
| MtVQ17 | AATGACAGTGTTCCACCTTC | CCAAAAGAAGTTGGTGAGAG |
| MtVQ18 | GCACCAACACCTATCAAAGT | TGGAGAGGACACTTCAACAT |
| MtVQ19 | AATGACAGTGTTCCACCTTC | CCAAAAGAAGTTGGTGAGAG |
| MtVQ20 | CTCCATTCCGTTAGAAAGC | TTGCTTTGTCACACCACTAC |
| MtVQ21 | CAGCTTACTGGATCACCTTC | CCATAATGAGCAGGTCTAGG |
| MtVQ22 | GCATCAAGAAGAGCACCTAC | AGGACGAAGAGGGTAAAAAG |
| MtVQ23 | GGTGGTTGCAGCAATAGTA | GTGCTTCTTCCATCATCAAC |
| MtVQ24 | GGTAAGCATTCTTCTGATCG | GGCACCATCTTCTCTCTAAG |
| MtVQ25 | AATTATGGTAACAACGTCGG | CCAAGGTCCTCATGTTATGT |
| MtVQ26 | GCTTGATACGTCAGCTTTTT | GATTCAAGAGTGGGAAAACA |
| MtVQ27 | AGGGTGGTTGTGAGAATATG | CATAGAATGCACTTGTTGGA |
| MtVQ28 | CCTCACAGGTAAAAACTCCA | GTCAGTGGAAAGCAAATAGC |
| MtVQ29 | ACTCCAATGTTGCTGAAACT | TTGAACGTGTTCACTTAACG |
| MtVQ30 | ACATCCCTGTCAGTGACTTC | GAGATAATATCCCCGGAAAC |
| MtVQ31 | ACATCCCTGTCAGTGACTTC | GAGATAATATCCCCGGAAAC |
| MtVQ32 | GCACCGATTACAAAGAGAAC | AATGTTGAAGGTGTGGTAGG |
