# Supplementary material for: Genome-wide identification and expression analysis of the VQ gene family in Cicer arietinum and Medicago truncatula
Source: PeerJ. 2020 Feb 4;8:e8471. doi: 10.7717/peerj.8471 (PMC7006518; doi:10.7717/peerj.8471)
Supplement: Table S2 [file peerj-08-8471-s008.docx]

| **Table S2 List of light responsive elements in the *VQ*** genes | | | |
| --- | --- | --- | --- |
| Gene name | *Cis*-elements | Number | Funcion |
| CaVQ13 | ACE | 1 | cis-acting element involved in light responsiveness |
| MtVQ12 | ACE | 1 | cis-acting element involved in light responsiveness |
| MtVQ22 | ACE | 1 | cis-acting element involved in light responsiveness |
| MtVQ24 | ACE | 1 | cis-acting element involved in light responsiveness |
| MtVQ25 | ACE | 1 | cis-acting element involved in light responsiveness |
| MtVQ28 | ACE | 1 | cis-acting element involved in light responsiveness |
| MtVQ29 | ACE | 1 | cis-acting element involved in light responsiveness |
| MtVQ29 | ACE | 1 | cis-acting element involved in light responsiveness |
| MtVQ30 | ACE | 1 | cis-acting element involved in light responsiveness |
| MtVQ5 | ACE | 2 | cis-acting element involved in light responsiveness |
| MtVQ6 | ACE | 1 | cis-acting element involved in light responsiveness |
| CaVQ10 | G-Box | 2 | cis-acting regulatory element involved in light responsiveness |
| CaVQ11 | G-box | 2 | cis-acting regulatory element involved in light responsiveness |
| CaVQ12 | G-box | 2 | cis-acting regulatory element involved in light responsiveness |
| CaVQ2 | G-Box | 1 | cis-acting regulatory element involved in light responsiveness |
| CaVQ5 | G-box | 1 | cis-acting regulatory element involved in light responsiveness |
| CaVQ6 | G-box | 1 | cis-acting regulatory element involved in light responsiveness |
| CaVQ8 | G-Box | 3 | cis-acting regulatory element involved in light responsiveness |
| CaVQ9 | G-Box | 2 | cis-acting regulatory element involved in light responsiveness |
| MtVQ01 | G-Box | 11 | cis-acting regulatory element involved in light responsiveness |
| MtVQ10 | G-box | 2 | cis-acting regulatory element involved in light responsiveness |
| MtVQ11 | G-Box | 5 | cis-acting regulatory element involved in light responsiveness |
| MtVQ14 | G-box | 1 | cis-acting regulatory element involved in light responsiveness |
| MtVQ15 | G-box | 1 | cis-acting regulatory element involved in light responsiveness |
| MtVQ16 | G-Box | 1 | cis-acting regulatory element involved in light responsiveness |
| MtVQ17 | G-Box | 2 | cis-acting regulatory element involved in light responsiveness |
| MtVQ18 | G-Box | 3 | cis-acting regulatory element involved in light responsiveness |
| MtVQ19 | G-Box | 3 | cis-acting regulatory element involved in light responsiveness |
| MtVQ2 | G-box | 1 | cis-acting regulatory element involved in light responsiveness |
| MtVQ20 | G-Box | 5 | cis-acting regulatory element involved in light responsiveness |
| MtVQ21 | G-Box | 3 | cis-acting regulatory element involved in light responsiveness |
| MtVQ22 | G-box | 3 | cis-acting regulatory element involved in light responsiveness |
| MtVQ23 | G-Box | 4 | cis-acting regulatory element involved in light responsiveness |
| MtVQ25 | G-Box | 2 | cis-acting regulatory element involved in light responsiveness |
| MtVQ26 | G-box | 1 | cis-acting regulatory element involved in light responsiveness |
| MtVQ27 | G-Box | 3 | cis-acting regulatory element involved in light responsiveness |
| MtVQ28 | G-Box | 5 | cis-acting regulatory element involved in light responsiveness |
| MtVQ29 | G-Box | 2 | cis-acting regulatory element involved in light responsiveness |
| MtVQ3 | G-Box | 7 | cis-acting regulatory element involved in light responsiveness |
| MtVQ30 | G-Box | 2 | cis-acting regulatory element involved in light responsiveness |
| MtVQ31 | G-box | 1 | cis-acting regulatory element involved in light responsiveness |
| MtVQ32 | G-box | 1 | cis-acting regulatory element involved in light responsiveness |
| MtVQ4 | G-Box | 4 | cis-acting regulatory element involved in light responsiveness |
| MtVQ5 | G-box | 2 | cis-acting regulatory element involved in light responsiveness |
| MtVQ6 | G-Box | 5 | cis-acting regulatory element involved in light responsiveness |
| MtVQ7 | G-Box | 2 | cis-acting regulatory element involved in light responsiveness |
| MtVQ9 | G-Box | 1 | cis-acting regulatory element involved in light responsiveness |
| CaVQ13 | 3-AF1 binding site | 2 | light responsive element |
| CaVQ14 | 3-AF1 binding site | 1 | light responsive element |
| CaVQ15 | 3-AF1 binding site | 2 | light responsive element |
| CaVQ17 | 3-AF1 binding site | 3 | light responsive element |
| CaVQ19 | 3-AF1 binding site | 3 | light responsive element |
| CaVQ6 | 3-AF1 binding site | 1 | light responsive element |
| CaVQ7 | 3-AF1 binding site | 1 | light responsive element |
| MtVQ11 | 3-AF1 binding site | 1 | light responsive element |
| MtVQ9 | 3-AF1 binding site | 1 | light responsive element |
| MtVQ10 | 4cl-CMA2b | 1 | light responsive element |
| MtVQ21 | AAAC-motif | 1 | light responsive element |
| MtVQ7 | AAAC-motif | 1 | light responsive element |
| CaVQ10 | AE-box | 2 | part of a module for light response |
| CaVQ12 | AE-box | 1 | part of a module for light response |
| CaVQ2 | AE-box | 2 | part of a module for light response |
| CaVQ3 | AE-box | 1 | part of a module for light response |
| CaVQ5 | AE-box | 1 | part of a module for light response |
| CaVQ7 | AE-box | 1 | part of a module for light response |
| MtVQ1 | AE-box | 2 | part of a module for light response |
| MtVQ13 | AE-box | 1 | part of a module for light response |
| MtVQ15 | AE-box | 2 | part of a module for light response |
| MtVQ2 | AE-box | 1 | part of a module for light response |
| MtVQ20 | AE-box | 1 | part of a module for light response |
| MtVQ21 | AE-box | 1 | part of a module for light response |
| MtVQ22 | AE-box | 2 | part of a module for light response |
| MtVQ23 | AE-box | 1 | part of a module for light response |
| MtVQ26 | AE-box | 1 | part of a module for light response |
| MtVQ32 | AE-box | 1 | part of a module for light response |
| MtVQ5 | AE-box | 1 | part of a module for light response |
| MtVQ8 | AE-box | 2 | part of a module for light response |
| CaVQ11 | ATC-motif | 1 | part of a conserved DNA module involved in light responsiveness |
| CaVQ4 | ATC-motif | 1 | part of a conserved DNA module involved in light responsiveness |
| MtVQ15 | ATC-motif | 1 | part of a conserved DNA module involved in light responsiveness |
| MtVQ23 | ATC-motif | 1 | part of a conserved DNA module involved in light responsiveness |
| MtVQ30 | ATC-motif | 1 | part of a conserved DNA module involved in light responsiveness |
| CaVQ17 | ATCT-motif | 1 | part of a conserved DNA module involved in light responsiveness |
| CaVQ18 | ATCT-motif | 2 | part of a conserved DNA module involved in light responsiveness |
| CaVQ19 | ATCT-motif | 1 | part of a conserved DNA module involved in light responsiveness |
| CaVQ8 | ATCT-motif | 1 | part of a conserved DNA module involved in light responsiveness |
| MtVQ12 | ATCT-motif | 1 | part of a conserved DNA module involved in light responsiveness |
| MtVQ17 | ATCT-motif | 1 | part of a conserved DNA module involved in light responsiveness |
| MtVQ20 | ATCT-motif | 2 | part of a conserved DNA module involved in light responsiveness |
| MtVQ21 | ATCT-motif | 1 | part of a conserved DNA module involved in light responsiveness |
| MtVQ4 | ATCT-motif | 1 | part of a conserved DNA module involved in light responsiveness |
| MtVQ9 | ATCT-motif | 1 | part of a conserved DNA module involved in light responsiveness |
| CaVQ1 | Box 4 | 3 | part of a conserved DNA module involved in light responsiveness |
| CaVQ10 | Box 4 | 7 | part of a conserved DNA module involved in light responsiveness |
| CaVQ11 | Box 4 | 6 | part of a conserved DNA module involved in light responsiveness |
| CaVQ12 | Box 4 | 3 | part of a conserved DNA module involved in light responsiveness |
| CaVQ13 | Box 4 | 4 | part of a conserved DNA module involved in light responsiveness |
| CaVQ14 | Box 4 | 1 | part of a conserved DNA module involved in light responsiveness |
| CaVQ15 | Box 4 | 4 | part of a conserved DNA module involved in light responsiveness |
| CaVQ17 | Box 4 | 3 | part of a conserved DNA module involved in light responsiveness |
| CaVQ19 | Box 4 | 3 | part of a conserved DNA module involved in light responsiveness |
| CaVQ2 | Box 4 | 7 | part of a conserved DNA module involved in light responsiveness |
| CaVQ3 | Box 4 | 8 | part of a conserved DNA module involved in light responsiveness |
| CaVQ4 | Box 4 | 2 | part of a conserved DNA module involved in light responsiveness |
| CaVQ5 | Box 4 | 6 | part of a conserved DNA module involved in light responsiveness |
| CaVQ6 | Box 4 | 9 | part of a conserved DNA module involved in light responsiveness |
| CaVQ7 | Box 4 | 15 | part of a conserved DNA module involved in light responsiveness |
| CaVQ8 | Box 4 | 4 | part of a conserved DNA module involved in light responsiveness |
| CaVQ9 | Box 4 | 3 | part of a conserved DNA module involved in light responsiveness |
| MtVQ1 | Box 4 | 2 | part of a conserved DNA module involved in light responsiveness |
| MtVQ10 | Box 4 | 1 | part of a conserved DNA module involved in light responsiveness |
| MtVQ11 | Box 4 | 3 | part of a conserved DNA module involved in light responsiveness |
| MtVQ12 | Box 4 | 4 | part of a conserved DNA module involved in light responsiveness |
| MtVQ13 | Box 4 | 1 | part of a conserved DNA module involved in light responsiveness |
| MtVQ14 | Box 4 | 3 | part of a conserved DNA module involved in light responsiveness |
| MtVQ15 | Box 4 | 2 | part of a conserved DNA module involved in light responsiveness |
| MtVQ16 | Box 4 | 1 | part of a conserved DNA module involved in light responsiveness |
| MtVQ17 | Box 4 | 3 | part of a conserved DNA module involved in light responsiveness |
| MtVQ18 | Box 4 | 3 | part of a conserved DNA module involved in light responsiveness |
| MtVQ19 | Box 4 | 1 | part of a conserved DNA module involved in light responsiveness |
| MtVQ2 | Box 4 | 5 | part of a conserved DNA module involved in light responsiveness |
| MtVQ20 | Box 4 | 2 | part of a conserved DNA module involved in light responsiveness |
| MtVQ21 | Box 4 | 5 | part of a conserved DNA module involved in light responsiveness |
| MtVQ22 | Box 4 | 8 | part of a conserved DNA module involved in light responsiveness |
| MtVQ23 | Box 4 | 6 | part of a conserved DNA module involved in light responsiveness |
| MtVQ24 | Box 4 | 1 | part of a conserved DNA module involved in light responsiveness |
| MtVQ25 | Box 4 | 3 | part of a conserved DNA module involved in light responsiveness |
| MtVQ26 | Box 4 | 3 | part of a conserved DNA module involved in light responsiveness |
| MtVQ27 | Box 4 | 3 | part of a conserved DNA module involved in light responsiveness |
| MtVQ28 | Box 4 | 2 | part of a conserved DNA module involved in light responsiveness |
| MtVQ29 | Box 4 | 5 | part of a conserved DNA module involved in light responsiveness |
| MtVQ3 | Box 4 | 4 | part of a conserved DNA module involved in light responsiveness |
| MtVQ30 | Box 4 | 3 | part of a conserved DNA module involved in light responsiveness |
| MtVQ31 | Box 4 | 1 | part of a conserved DNA module involved in light responsiveness |
| MtVQ32 | Box 4 | 2 | part of a conserved DNA module involved in light responsiveness |
| MtVQ4 | Box 4 | 1 | part of a conserved DNA module involved in light responsiveness |
| MtVQ6 | Box 4 | 5 | part of a conserved DNA module involved in light responsiveness |
| MtVQ7 | Box 4 | 2 | part of a conserved DNA module involved in light responsiveness |
| MtVQ8 | Box 4 | 6 | part of a conserved DNA module involved in light responsiveness |
| MtVQ9 | Box 4 | 4 | part of a conserved DNA module involved in light responsiveness |
| CaVQ17 | chs-CMA1a | 1 | part of a light responsive element |
| CaVQ19 | chs-CMA1a | 1 | part of a light responsive element |
| CaVQ3 | chs-CMA1a | 2 | part of a light responsive element |
| CaVQ4 | chs-CMA1a | 2 | part of a light responsive element |
| MtVQ11 | chs-CMA1a | 1 | part of a light responsive element |
| MtVQ17 | chs-CMA1a | 1 | part of a light responsive element |
| MtVQ18 | chs-CMA1a | 1 | part of a light responsive element |
| MtVQ27 | chs-CMA1a | 1 | part of a light responsive element |
| MtVQ3 | chs-CMA1a | 1 | part of a light responsive element |
| MtVQ32 | chs-CMA1a | 2 | part of a light responsive element |
| CaVQ18 | chs-CMA2a | 1 | part of a light responsive element |
| MtVQ15 | chs-CMA2a | 1 | part of a light responsive element |
| MtVQ4 | chs-CMA2a | 1 | part of a light responsive element |
| MtVQ10 | chs-Unit 1 m1 | 1 | part of a light responsive element |
| CaVQ10 | GA-motif | 1 | part of a light responsive element |
| CaVQ12 | GA-motif | 1 | part of a light responsive element |
| CaVQ15 | GA-motif | 2 | part of a light responsive element |
| CaVQ17 | GA-motif | 1 | part of a light responsive element |
| CaVQ19 | GA-motif | 1 | part of a light responsive element |
| CaVQ2 | GA-motif | 1 | part of a light responsive element |
| MtVQ10 | GA-motif | 1 | part of a light responsive element |
| MtVQ13 | GA-motif | 1 | part of a light responsive element |
| MtVQ15 | GA-motif | 1 | part of a light responsive element |
| MtVQ16 | GA-motif | 1 | part of a light responsive element |
| MtVQ23 | GA-motif | 1 | part of a light responsive element |
| MtVQ26 | GA-motif | 1 | part of a light responsive element |
| MtVQ31 | GA-motif | 1 | part of a light responsive element |
| MtVQ4 | GA-motif | 1 | part of a light responsive element |
| MtVQ5 | GA-motif | 1 | part of a light responsive element |
| MtVQ7 | GA-motif | 2 | part of a light responsive element |
| CaVQ6 | Gap-box | 1 | part of a light responsive element |
| MtVQ16 | Gap-box | 1 | part of a light responsive element |
| MtVQ19 | Gap-box | 1 | part of a light responsive element |
| CaVQ15 | GATA-motif | 1 | part of a light responsive element |
| CaVQ17 | GATA-motif | 3 | part of a light responsive element |
| CaVQ19 | GATA-motif | 3 | part of a light responsive element |
| CaVQ8 | GATA-motif | 1 | part of a light responsive element |
| MtVQ10 | GATA-motif | 1 | part of a light responsive element |
| MtVQ11 | GATA-motif | 1 | part of a light responsive element |
| MtVQ12 | GATA-motif | 1 | part of a light responsive element |
| MtVQ19 | GATA-motif | 4 | part of a light responsive element |
| MtVQ2 | GATA-motif | 2 | part of a light responsive element |
| MtVQ25 | GATA-motif | 1 | part of a light responsive element |
| MtVQ27 | GATA-motif | 1 | part of a light responsive element |
| MtVQ30 | GATA-motif | 1 | part of a light responsive element |
| MtVQ7 | GATA-motif | 1 | part of a light responsive element |
| MtVQ9 | GATA-motif | 1 | part of a light responsive element |
| CaVQ10 | G-Box | 2 | cis-acting regulatory element involved in light responsiveness |
| CaVQ11 | G-box | 2 | cis-acting regulatory element involved in light responsiveness |
| CaVQ12 | G-box | 2 | cis-acting regulatory element involved in light responsiveness |
| CaVQ2 | G-Box | 1 | cis-acting regulatory element involved in light responsiveness |
| CaVQ5 | G-box | 1 | cis-acting regulatory element involved in light responsiveness |
| CaVQ6 | G-box | 1 | cis-acting regulatory element involved in light responsiveness |
| CaVQ8 | G-Box | 3 | cis-acting regulatory element involved in light responsiveness |
| CaVQ9 | G-Box | 2 | cis-acting regulatory element involved in light responsiveness |
| MtVQ1 | G-Box | 11 | cis-acting regulatory element involved in light responsiveness |
| MtVQ10 | G-box | 2 | cis-acting regulatory element involved in light responsiveness |
| MtVQ11 | G-Box | 5 | cis-acting regulatory element involved in light responsiveness |
| MtVQ14 | G-box | 1 | cis-acting regulatory element involved in light responsiveness |
| MtVQ15 | G-box | 1 | cis-acting regulatory element involved in light responsiveness |
| MtVQ16 | G-Box | 1 | cis-acting regulatory element involved in light responsiveness |
| MtVQ17 | G-Box | 2 | cis-acting regulatory element involved in light responsiveness |
| MtVQ18 | G-Box | 3 | cis-acting regulatory element involved in light responsiveness |
| MtVQ19 | G-Box | 3 | cis-acting regulatory element involved in light responsiveness |
| MtVQ2 | G-box | 1 | cis-acting regulatory element involved in light responsiveness |
| MtVQ20 | G-Box | 5 | cis-acting regulatory element involved in light responsiveness |
| MtVQ21 | G-Box | 3 | cis-acting regulatory element involved in light responsiveness |
| MtVQ22 | G-box | 3 | cis-acting regulatory element involved in light responsiveness |
| MtVQ23 | G-Box | 4 | cis-acting regulatory element involved in light responsiveness |
| MtVQ25 | G-Box | 2 | cis-acting regulatory element involved in light responsiveness |
| MtVQ26 | G-box | 1 | cis-acting regulatory element involved in light responsiveness |
| MtVQ27 | G-Box | 3 | cis-acting regulatory element involved in light responsiveness |
| MtVQ28 | G-Box | 5 | cis-acting regulatory element involved in light responsiveness |
| MtVQ29 | G-Box | 2 | cis-acting regulatory element involved in light responsiveness |
| MtVQ3 | G-Box | 7 | cis-acting regulatory element involved in light responsiveness |
| MtVQ30 | G-Box | 2 | cis-acting regulatory element involved in light responsiveness |
| MtVQ31 | G-box | 1 | cis-acting regulatory element involved in light responsiveness |
| MtVQ32 | G-box | 1 | cis-acting regulatory element involved in light responsiveness |
| MtVQ4 | G-Box | 4 | cis-acting regulatory element involved in light responsiveness |
| MtVQ5 | G-box | 2 | cis-acting regulatory element involved in light responsiveness |
| MtVQ6 | G-Box | 5 | cis-acting regulatory element involved in light responsiveness |
| MtVQ7 | G-Box | 2 | cis-acting regulatory element involved in light responsiveness |
| MtVQ9 | G-Box | 1 | cis-acting regulatory element involved in light responsiveness |
| CaVQ1 | GT1-motif | 2 | light responsive element |
| CaVQ10 | GT1-motif | 1 | light responsive element |
| CaVQ11 | GT1-motif | 2 | light responsive element |
| CaVQ13 | GT1-motif | 2 | light responsive element |
| CaVQ17 | GT1-motif | 2 | light responsive element |
| CaVQ19 | GT1-motif | 2 | light responsive element |
| CaVQ2 | GT1-motif | 1 | light responsive element |
| CaVQ4 | GT1-motif | 5 | light responsive element |
| CaVQ6 | GT1-motif | 1 | light responsive element |
| CaVQ7 | GT1-motif | 2 | light responsive element |
| CaVQ8 | GT1-motif | 2 | light responsive element |
| CaVQ9 | GT1-motif | 2 | light responsive element |
| MtVQ10 | GT1-motif | 1 | light responsive element |
| MtVQ16 | GT1-motif | 2 | light responsive element |
| MtVQ17 | GT1-motif | 3 | light responsive element |
| MtVQ18 | GT1-motif | 5 | light responsive element |
| MtVQ19 | GT1-motif | 1 | light responsive element |
| MtVQ2 | GT1-motif | 1 | light responsive element |
| MtVQ21 | GT1-motif | 3 | light responsive element |
| MtVQ22 | GT1-motif | 2 | light responsive element |
| MtVQ23 | GT1-motif | 1 | light responsive element |
| MtVQ24 | GT1-motif | 1 | light responsive element |
| MtVQ26 | GT1-motif | 1 | light responsive element |
| MtVQ27 | GT1-motif | 7 | light responsive element |
| MtVQ28 | GT1-motif | 3 | light responsive element |
| MtVQ29 | GT1-motif | 2 | light responsive element |
| MtVQ31 | GT1-motif | 4 | light responsive element |
| MtVQ32 | GT1-motif | 3 | light responsive element |
| MtVQ4 | GT1-motif | 1 | light responsive element |
| MtVQ5 | GT1-motif | 1 | light responsive element |
| MtVQ7 | GT1-motif | 1 | light responsive element |
| MtVQ8 | GT1-motif | 1 | light responsive element |
| CaVQ17 | GTGGC-motif | 2 | part of a light responsive element |
| CaVQ19 | GTGGC-motif | 1 | part of a light responsive element |
| CaVQ1 | I-box | 2 | part of a light responsive element |
| CaVQ10 | I-box | 1 | part of a light responsive element |
| CaVQ11 | I-box | 1 | part of a light responsive element |
| CaVQ15 | I-box | 1 | part of a light responsive element |
| CaVQ2 | I-box | 1 | part of a light responsive element |
| CaVQ4 | I-box | 1 | part of a light responsive element |
| CaVQ5 | I-box | 1 | part of a light responsive element |
| CaVQ8 | I-box | 1 | part of a light responsive element |
| CaVQ9 | I-box | 2 | part of a light responsive element |
| MtVQ10 | I-box | 1 | part of a light responsive element |
| MtVQ11 | I-box | 2 | part of a light responsive element |
| MtVQ15 | I-box | 1 | part of a light responsive element |
| MtVQ19 | I-box | 2 | part of a light responsive element |
| MtVQ2 | I-box | 1 | part of a light responsive element |
| MtVQ27 | I-box | 1 | part of a light responsive element |
| MtVQ32 | I-box | 1 | part of a light responsive element |
| MtVQ8 | I-box | 2 | part of a light responsive element |
| CaVQ10 | LAMP-element | 1 | part of a light responsive element |
| CaVQ17 | LAMP-element | 1 | part of a light responsive element |
| CaVQ19 | LAMP-element | 1 | part of a light responsive element |
| CaVQ7 | LAMP-element | 1 | part of a light responsive element |
| CaVQ9 | LAMP-element | 1 | part of a light responsive element |
| MtVQ14 | LAMP-element | 1 | part of a light responsive element |
| MtVQ21 | LAMP-element | 1 | part of a light responsive element |
| CaVQ10 | MRE | 1 | MYB binding site involved in light responsiveness |
| CaVQ11 | MRE | 2 | MYB binding site involved in light responsiveness |
| CaVQ12 | MRE | 1 | MYB binding site involved in light responsiveness |
| CaVQ14 | MRE | 1 | MYB binding site involved in light responsiveness |
| CaVQ15 | MRE | 1 | MYB binding site involved in light responsiveness |
| CaVQ2 | MRE | 1 | MYB binding site involved in light responsiveness |
| CaVQ3 | MRE | 1 | MYB binding site involved in light responsiveness |
| CaVQ4 | MRE | 1 | MYB binding site involved in light responsiveness |
| CaVQ5 | MRE | 1 | MYB binding site involved in light responsiveness |
| CaVQ7 | MRE | 1 | MYB binding site involved in light responsiveness |
| CaVQ8 | MRE | 1 | MYB binding site involved in light responsiveness |
| MtVQ14 | MRE | 1 | MYB binding site involved in light responsiveness |
| MtVQ15 | MRE | 1 | MYB binding site involved in light responsiveness |
| MtVQ18 | MRE | 1 | MYB binding site involved in light responsiveness |
| MtVQ20 | MRE | 1 | MYB binding site involved in light responsiveness |
| MtVQ22 | MRE | 1 | MYB binding site involved in light responsiveness |
| MtVQ24 | MRE | 1 | MYB binding site involved in light responsiveness |
| MtVQ26 | MRE | 1 | MYB binding site involved in light responsiveness |
| MtVQ27 | MRE | 1 | MYB binding site involved in light responsiveness |
| MtVQ28 | MRE | 1 | MYB binding site involved in light responsiveness |
| MtVQ3 | MRE | 1 | MYB binding site involved in light responsiveness |
| MtVQ31 | MRE | 1 | MYB binding site involved in light responsiveness |
| MtVQ4 | MRE | 2 | MYB binding site involved in light responsiveness |
| MtVQ25 | sbp-CMA1c | 1 | part of a light responsive element |
| CaVQ11 | Sp1 | 1 | light responsive element |
| MtVQ21 | Sp1 | 1 | light responsive element |
| MtVQ24 | Sp1 | 2 | light responsive element |
| MtVQ32 | Sp1 | 1 | light responsive element |
| CaVQ1 | TCCC-motif | 1 | part of a light responsive element |
| CaVQ8 | TCCC-motif | 2 | part of a light responsive element |
| CaVQ9 | TCCC-motif | 1 | part of a light responsive element |
| MtVQ16 | TCCC-motif | 1 | part of a light responsive element |
| MtVQ26 | TCCC-motif | 1 | part of a light responsive element |
| MtVQ3 | TCCC-motif | 2 | part of a light responsive element |
| MtVQ32 | TCCC-motif | 1 | part of a light responsive element |
| CaVQ1 | TCT-motif | 1 | part of a light responsive element |
| CaVQ11 | TCT-motif | 3 | part of a light responsive element |
| CaVQ13 | TCT-motif | 2 | part of a light responsive element |
| CaVQ14 | TCT-motif | 3 | part of a light responsive element |
| CaVQ15 | TCT-motif | 2 | part of a light responsive element |
| CaVQ17 | TCT-motif | 3 | part of a light responsive element |
| CaVQ18 | TCT-motif | 1 | part of a light responsive element |
| CaVQ19 | TCT-motif | 3 | part of a light responsive element |
| CaVQ2 | TCT-motif | 1 | part of a light responsive element |
| CaVQ4 | TCT-motif | 1 | part of a light responsive element |
| CaVQ5 | TCT-motif | 1 | part of a light responsive element |
| CaVQ6 | TCT-motif | 1 | part of a light responsive element |
| CaVQ9 | TCT-motif | 2 | part of a light responsive element |
| MtVQ1 | TCT-motif | 1 | part of a light responsive element |
| MtVQ10 | TCT-motif | 2 | part of a light responsive element |
| MtVQ12 | TCT-motif | 1 | part of a light responsive element |
| MtVQ14 | TCT-motif | 1 | part of a light responsive element |
| MtVQ16 | TCT-motif | 2 | part of a light responsive element |
| MtVQ18 | TCT-motif | 1 | part of a light responsive element |
| MtVQ19 | TCT-motif | 1 | part of a light responsive element |
| MtVQ22 | TCT-motif | 3 | part of a light responsive element |
| MtVQ25 | TCT-motif | 1 | part of a light responsive element |
| MtVQ27 | TCT-motif | 1 | part of a light responsive element |
| MtVQ28 | TCT-motif | 1 | part of a light responsive element |
| MtVQ29 | TCT-motif | 1 | part of a light responsive element |
| MtVQ32 | TCT-motif | 1 | part of a light responsive element |
